# Preliminary Study on the Geochemical Characterization of Viticis Fructus Cuticular Waxes: From Latitudinal Variation to Origin Authentication

**DOI:** 10.3390/ijms26157293

**Published:** 2025-07-28

**Authors:** Yiqing Luo, Min Guo, Lei Hu, Jiaxin Yang, Junyu Xu, Muhammad Rafiq, Ying Wang, Chunsong Cheng, Shaohua Zeng

**Affiliations:** 1 Guangdong Provincial Key Laboratory of Applied Botany, South China Botanical Garden, Chinese Academy of Sciences, Guangzhou 510650, China; yiqingluo1994@163.com (Y.L.);; 2 Jiangxi Key Laboratory for Sustainable Utilization of Chinese Materia Medica Resources, Lushan Botanical Garden, Chinese Academy of Sciences, Jiujiang 332900, China; 3 Lushan Xinglin Institute for Medicinal Plants, Jiujiang Xinglin Key Laboratory for Traditional Chinese Medicines, Lushan, Jiujiang 332900, China; 4 School of Life Sciences, Nanchang University, Nanchang 330031, China

**Keywords:** Viticis Fructus, cuticular waxes, origin identification, origin authentication, biomarkers

## Abstract

*Viticis Fructus* (VF), a fruit known for its unique flavor profile and various health benefits, demonstrates substantial quality variations depending on its area of production. Traditional methods of production area verification based on internal compound analysis are hampered by a number of technical limitations. This investigation systematically characterized the cuticular wax composition of VF sample from a diverse variety of production areas. Quantitative analyses were conducted to evaluate the spatial distribution patterns of the wax constituents. Significant regional variations were observed: Anhui sample exhibited the highest total wax content (21.39 μg/cm^2^), with n-alkanes dominating at 76.67%. High-latitude regions showed elevated triterpenoid acid levels, with maslinic acid (0.53 μg/cm^2^) and ursolic acid (0.34 μg/cm^2^) concentrations exceeding those of their low-latitude counterparts by four- and three-fold, respectively. Altitudinal influence manifested in long-chain alcohol accumulation, as triacontanol reached 0.87 μg/cm^2^ in high-altitude sample. Five key biomarkers demonstrated direct quality correlations: eicosanoic acid, n-triacontane, dotriacontanol, β-amyrin, and α-amyrin. This study established three novel origin identification protocols: single-component quantification, multi-component wax profiling, and wax ratio analysis. This work not only reveals the latitudinal dependence of VF wax composition, but also provides a scientific framework for geographical authentication. Our findings advance wax-based quality evaluation methodologies for fruit products, offering practical solutions for production area verification challenges in food raw materials.

## 1. Introduction

*Viticis Fructus* (VF), the dried ripe fruit of *Vitex rotundifolia* L. f. and *Vitex trifolia* L. (family Lamiaceae), is a traditional raw food material that is well-known for its unique flavor profile and various health benefits [1]. It has been widely used in daily diets to alleviate symptoms such as colds, headaches, migraines, and eye pain [2,3,4,5]. Modern pharmacological research has identified key flavonoids in VF, such as vitexin, which exhibits analgesic, anti-inflammatory, and antitumor activities, while rutin has been found to possess vasodilatory effects [6,7]. Additionally, due to its distinctive aroma, VF is commonly used as a food additive and flavoring agent, enhancing the taste of various dishes. In China, South Korea, and Japan, VF is also processed into tea, which is highly regarded for its unique fragrance and notable health benefits [8].

Both *Vitex rotundifolia* L. f. and *Vitex trifolia* L. are highly adaptable, being capable of thriving in challenging environments. These species can endure drought, alkalinity, high temperatures, and short-term frost [9]. They prefer sunny conditions and grow well in tropical and subtropical regions, particularly in coarse sandy soils, rocky terrains, and sandy grasslands. They are typically found near water bodies, such as beaches, lakesides, and riverbanks, where sunlight is abundant [10]. In China, they are primarily distributed across the provinces of Anhui, Shandong, Zhejiang, Jiangxi, Yunnan, and Hainan. Additionally, Vitex species are found in Japan, India, Myanmar, Thailand, Vietnam, Malaysia, Australia, and New Zealand [11].

The quality of VF varies significantly due to natural environmental factors, including climate, soil composition, and water quality [10,12,13]. These variations influence its chemical composition, affecting the levels of volatile oils, flavonoids, triterpenoids, sesquiterpenes, fatty acids, alkaloids, steroids, amino acids, and other bioactive compounds [14,15,16]. These constituents, alongside their distinctive flavor characteristics, directly contribute to their antitumor, antipyretic, analgesic, anti-inflammatory, antibacterial, antihypertensive, and antioxidant activities [17,18].

**Figure 1:**
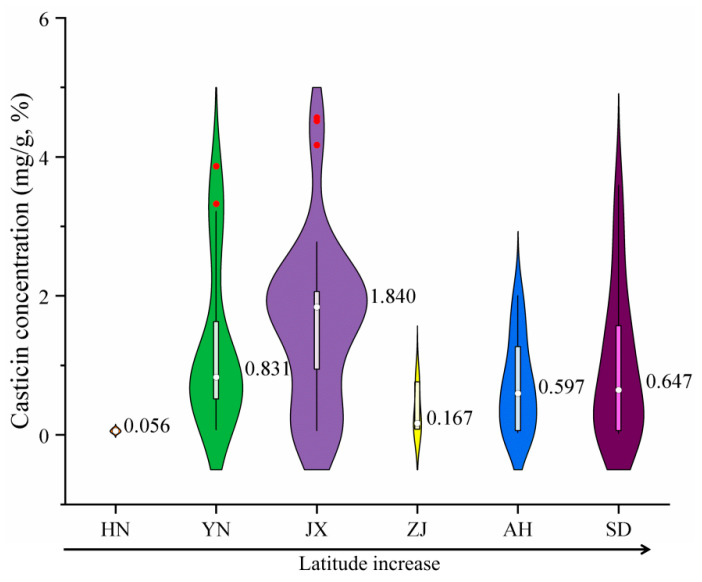
Internal casticin content of VF collected in Hainan, Yunnan, Jiangxi, Zhejiang, Anhui, and Shandong Provinces (in the violin plot, the width of the violin represents the density of the data, thin lines indicate 95% confidence intervals, white bar-shaped regions indicate interquartile ranges, red dots indicate outliers, and white dots indicate means).

Current research on VF commonly utilizes gas chromatography–mass spectrometry (GC-MS) to analyze chemical profiles across different production areas, with a primary focus on casticin, one of its most significant metabolites [19,20,21]. Figure 1 presents the casticin content of VF sample from various production areas. The results indicate that there is no clear monotonic correlation between casticin levels and the latitude of the production area.

The main bioactive components of VF, such as casticin, are plant secondary metabolites. Their synthesis is highly influenced by growth stage, plant health, and climatic conditions, leading to significant temporal and individual variations that can reduce the reliability of production area discrimination. These metabolites are located inside the fruit and are susceptible to sample processing (e.g., drying, oxidation, variations in extraction solvents), potentially introducing analytical errors. Moreover, differences between various producing areas may primarily manifest in content rather than composition, making it difficult to establish a distinct “fingerprint” for identification purposes. Consequently, relying solely on casticin content to determine the production area of VF is challenging [22,23].

Cuticular wax is a lipid secreted by the outer layer of plant epidermal cells, primarily consisting of long-chain fatty acids, primary alcohols, and esters [24,25,26,27]. These waxes form a protective layer on the plant cuticula, shielding them from physical and chemical damage, minimizing water loss, and providing resistance against UV radiation, extreme temperature fluctuations, and various diseases and pests [28,29,30,31]. Due to genetic and environmental variations, plants from different production areas exhibit distinct wax composition profiles. As a result, the wax composition of the plant cuticula is species-specific, with variations in both the types and proportions of the components [32,33,34]. Although environmental variations may slightly affect wax thickness or content, the relative proportions of key components are more strongly influenced by geographical origin and are less prone to short-term fluctuations. Crucially, wax composition—including carbon chain distribution and branching patterns—reflects long-term environmental adaptation, thereby serving as a robust indicator of region-specific ecological traits. Furthermore, non-destructive detection methods are typically employed during wax analysis, minimizing experimental errors. For numerous plants (grapes, tea leaves, and Chinese medicinal herbs), surface wax has demonstrated high efficacy in discriminating production areas due to its environmentally linked chemical signature [35,36,37,38,39]. Compared to secondary metabolites like casticin in VF, surface wax exhibits higher chemical stability, lower susceptibility to environmental fluctuations, and greater compositional diversity [40,41]. As a result, it provides a more consistent representation of geographical traits, making it a more accurate marker for production area identification [42].

This study investigates the wax composition of VF from different production regions, examining its relationship with environmental factors. The primary objective is to accurately determine the production area of VF. A total of 22 wax components were identified across major production areas. Among these, specific waxes from extreme production regions—Shandong (high latitude) and Yunnan (high altitude)—were highlighted, facilitating the identification of VF from these locations. Furthermore, correlations were established between the content of key wax components (hentriacontane, n-tritriacontane, n-pentatriacontane, dotriacontane, and n-tetratriacontane) and the latitude of other major production areas. Based on these correlations, three identification methods were developed: the multi-component wax method, the single-component wax method, and the wax ratio method. These approaches provide a comprehensive framework for analyzing the relationship between VF wax composition and latitude, enabling the precise identification of VF from unknown production areas.

Additionally, this study explored the relationship between wax components and internal metabolite compounds, identifying key waxes (eicosanoic acid, n-triacontane, dotriacontanol, beta-amyrin, and alpha-amyrin) that are directly correlated with VF quality. The findings from this study offer a scientific foundation for the rapid, accurate, and minimally invasive identification of VF production areas. Moreover, this research reveals the intrinsic connection between cuticular wax composition and production area, promoting the application of wax analysis in the quality assessment of fruit products.

## 2. Results and Discussion

### 2.1. Chemical Composition and Content Analysis of VF Cuticular Waxes

**Figure 2:**
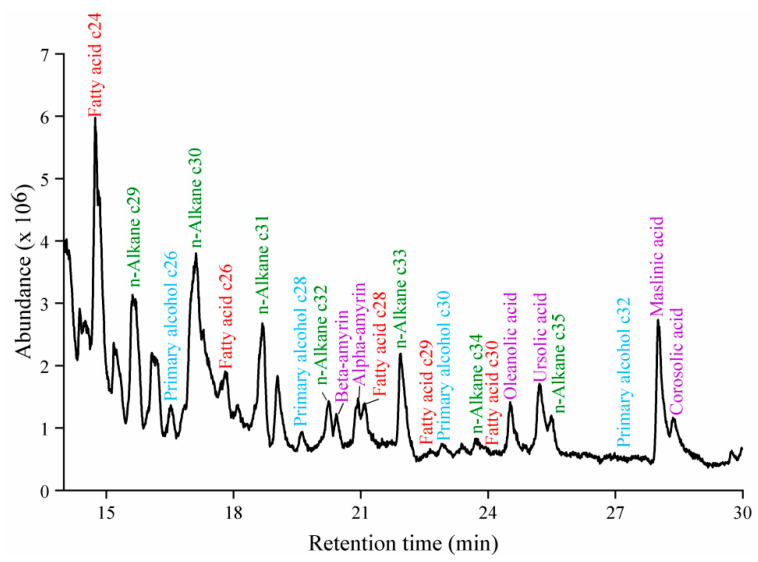
GC-MS chromatogram of VF wax (red represents fatty acids, green represents n-alkanes, blue represents primary alcohols, and purple represents triterpenoids).

Cuticular wax was extracted from VF sample collected from various production areas and analyzed using GC-MS. The wax components were identified by comparing the obtained spectra with the NIST database (Figure 2). Fatty acids, n-alkanes, primary alcohols, and triterpenoids were screened. Based on their content levels, twenty-two wax components were selected for further analysis, as detailed in Table 1.

**Figure 3:**
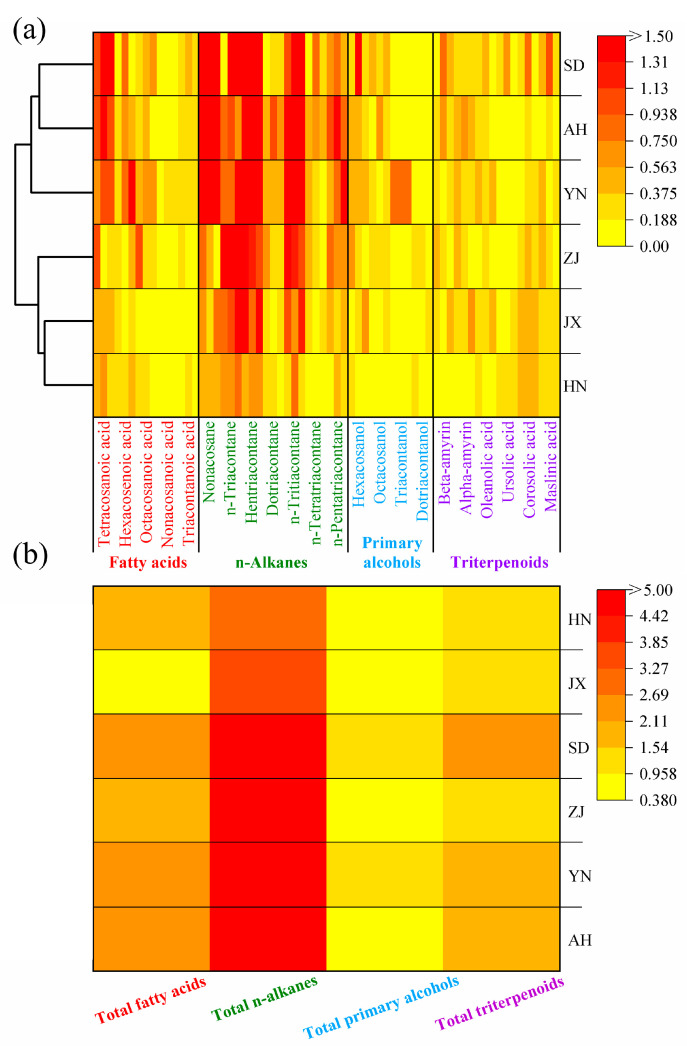
(**a**) Wax content heat-map of VF cuticular waxes from different production areas (left side shows clustering groups). (**b**) Wax content heat-map of the four main categories of VF cuticular waxes from different production areas.

**Figure 4:**
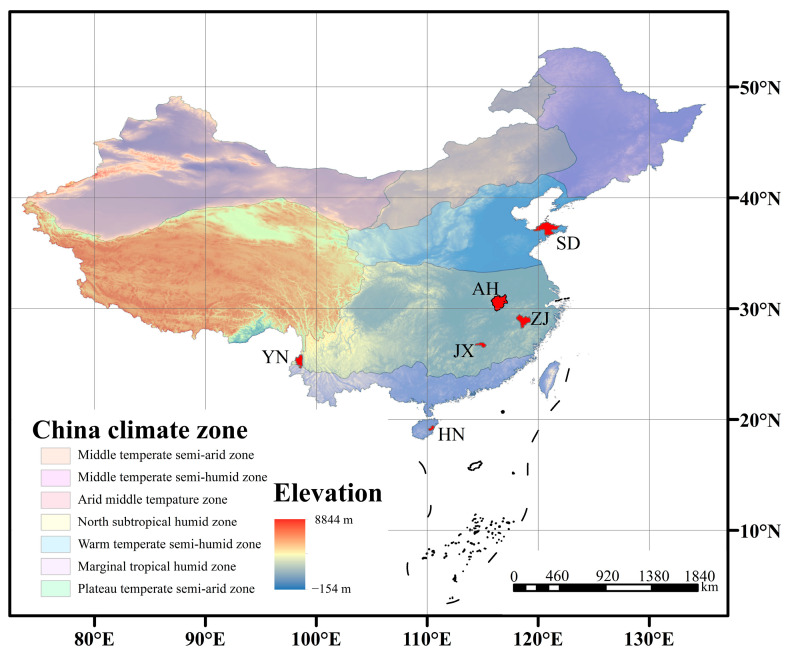
Schematic diagram of climate zones and altitudes from different VF production areas (the red patch indicates the area of the city where the production area is located).

Based on the GC-MS results and the cuticular area measurements of VF, wax content from various production areas was obtained. A heat-map was generated to visually present the data (Figure 3a). The wax content ranged from low to high, with color blocks transitioning from light yellow to deep red. The overall color for HN was the lightest, indicating the lowest wax content, followed by JX, ZJ, YN, SD, and AH. The total wax content from AH was 21.39 μg/cm^2^, approximately 3.3 times higher than HN (6.42 μg/cm^2^). The low wax content from HN is attributed to its tropical monsoon climate and low latitude, which are less favorable for wax accumulation on VF (Figure 4).

The low wax content in HN aligns with its tropical monsoon climate (24~26 °C annual temperature, 2000~2500 mm annual precipitation; Table 2). High humidity and temperatures in HN may downregulate wax biosynthesis pathways, as cuticular waxes primarily function to prevent water loss under arid conditions [43,44,45,46]. The laterite and red soils in HN (pH 5.0~6.5) are acidic and nutrient-leached, which may further limit the allocation of resources to wax synthesis, as plants prioritize growth over stress-resistant wax production in such high-resource (water, temperature) environments [47,48].

In contrast, AH showed the highest wax content, likely due to its cooler climate (15~17 °C) and intermediate precipitation (1300–1500 mm) [49,50]. The yellow-brown and paddy soils in AH (pH 5.5~7.0) are more neutral and fertile, providing sufficient nutrients for wax biosynthesis. The moderate precipitation creates a balance: plants need to retain water without excessive wax accumulation, but the cooler temperatures slow metabolic rates, allowing for longer-term wax deposition. This environment explains why AH had the highest n-alkane content (16.4 μg/cm^2^, 76.67% of total wax), as n-alkanes are key structural components for reducing transpiration in temperate climates with seasonal moisture variation.

The YN area, despite being at a relatively low latitude (25.02° N), diverged from other low-latitude sites (e.g., HN) due to its high altitude (1600~1900 m). The volcanic ash soil in YN is rich in minerals (e.g., silica, potassium), which may enhance enzyme activity in wax biosynthesis. The cooler temperatures (14–16 °C) at high altitudes, combined with intense UV radiation, likely induce the synthesis of UV-protective wax components, explaining its distinct wax profile compared to HN [51,52,53].

Cluster analysis of the wax content across all areas revealed that JX and HN clustered together, while SD and AH formed another group. SD and AH share similar latitudinal positions and environmental conditions, which explains the similarity in their wax component content. Interestingly, the classification of JX and HN into the same group does not align with their latitudinal relationship. Although YN is geographically closer to HN, it did not cluster with HN. This discrepancy is likely due to the higher altitude of YN, which creates unique climatic conditions that increase wax accumulation on the VF cuticle.

To further visualize the content of wax categories, a heat-map was created for four primary categories (Figure 3b). The darkest color block corresponded to n-alkanes, indicating that n-alkanes had the highest content among all wax categories. In AH, which had the highest total wax content, n-alkanes measured 16.4 μg/cm^2^, accounting for 76.67% of the total wax. In contrast, the content of n-alkanes from HN, YN, JX, ZJ, and SD were 2.75, 10.10, 5.00, 6.42, and 10.50 μg/cm^2^, respectively, accounting for 42.8%, 61.8%, 54.9%, 61.8%, and 63.0% of the total wax content from each area (Table 3).

### 2.2. Special Production Area Priority Identification and Screening of VF

**Figure 5:**
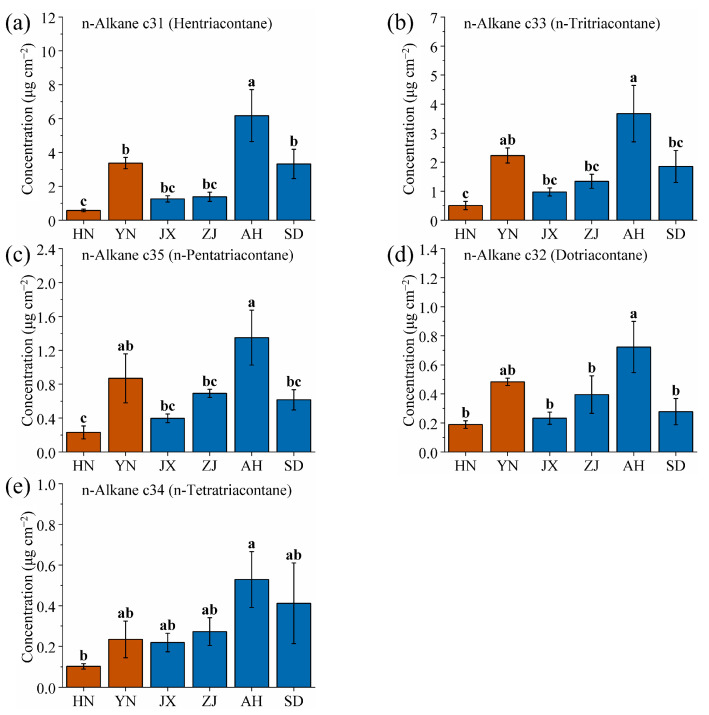
Comparison of (**a**) hentriacontane, (**b**) n-tritriacontane, (**c**) n-pentatriacontane, (**d**) dotriacontane, and (**e**) n-tetratriacontane wax contents of VF from six production areas (HN, YN, JX, ZJ, AH and SD). Different lowercase letters indicate significant differences, *p* < 0.05, Duncan’s HSD test.

Figure 5a–e present column charts illustrating the content of five kinds of n-alkane wax on the cuticle of VF from various production areas (significance analysis as shown in Table S1). The figures are arranged in descending order based on wax content. The horizontal axis indicates increasing latitude from left to right, with deep brown columns representing dried fruits of *Vitex trifolia* L. and deep blue columns representing dried fruits of *Vitex rotundifolia* L. f. Among all wax components, hentriacontane exhibits the highest content. Across all areas, the average content of hentriacontane is 17.6%, which is notably high. The area with the highest hentriacontane content is AH (6.18 mg/cm^2^), accounting for 28.88% of the total wax content. In contrast, the area with the lowest hentriacontane content is HN (0.58 mg/cm^2^), accounting for 9.00% of the total wax content. The content from AH is approximately ten times higher than that from HN, highlighting a significant difference between these two areas (Table 4).

For other high-content waxes such as n-tritriacontane, n-pentatriacontane, dotriacontane, and n-tetratriacontane, AH consistently exhibits the highest content, with values of 3.67, 1.35, 0.72, and 0.53 mg/cm^2^, respectively, accounting for 17.17%, 6.31%, 3.38%, and 2.47% of the total waxes. Conversely, HN consistently exhibits the lowest content, with values of 0.50, 0.23, 0.19, and 0.10 mg/cm^2^, respectively, accounting for 7.85%, 3.60%, 2.94%, and 1.59% of the total wax content from that area (Table 4).

The ranking of the wax content across various production areas demonstrates similarities, particularly from HN, JX, ZJ, and AH. From these four areas, wax content exhibits a monotonic correlation with latitude. However, YN and SD are quite special. The main reason is the high altitude of the YN area, in contrast to the relatively low altitudes of the other production areas. Additionally, SD is located in northern China and is characterized by a temperate monsoon climate, which results in less precipitation and sunlight compared to the other areas [54,55,56]. The growth environments of *Vitex rotundifolia* L. f. and *Vitex trifolia* L. from YN and SD differ significantly from the other four areas, leading to variations in the synthesis and accumulation of waxes on VF. Consequently, this study proposes a method to identify VF from YN and SD. If VFs from YN and SD are present, they will be prioritized for identification and marking. If VF from YN or SD are not present, further identification will be based on the monotonic correlation between wax content and latitude. To achieve this goal, this study aims to find the specific wax components that distinguish VF grown in YN and SD.

**Figure 6:**
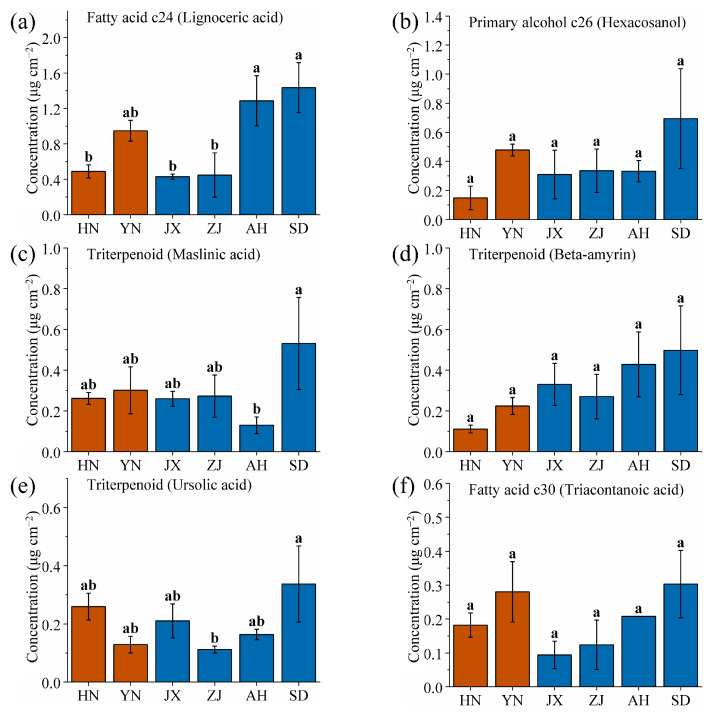
Comparison of (**a**) lignoceric acid, (**b**) hexacosanol, (**c**) maslinic acid, (**d**) beta-amyrin, (**e**) ursolic acid, and (**f**) triacontanoic acid wax contents of VF from six production areas (HN, YN, JX, ZJ, AH, and SD). Different lowercase letters indicate significant differences, *p* < 0.05, Duncan’s HSD test.

This study identified six wax components with the highest content in SD: lignoceric acid, hexacosanol, maslinic acid, beta-amyrin, ursolic acid, and triacontanoic acid. The aim was to identify SD-specific wax components to aid in prioritizing identification of the SD production area (Figure 6, significance analysis as shown in Table S2). Among these six components, three are triterpenoids, suggesting that high-latitude, low-precipitation environments favor the synthesis and accumulation of triterpenoid waxes by VF. Meanwhile, the brown soil in SD (pH 6.5~7.5) is alkaline, which may upregulate genes involved in triterpenoid biosynthesis. Triterpenoids act as antioxidants and stress protectants, playing critical roles in plant adaption to cold winters (frost-free period 200~220 days) and drought conditions (low precipitation) [57,58,59]. The low humidity and large temperature fluctuations in SD further select for triterpenoids, which stabilize cell membranes under thermal stress [60,61]. The contents of lignoceric acid, hexacosanol, beta-amyrin, and triacontanoic acid from SD did not differ significantly from other areas, and they were therefore not considered SD-specific wax components (Figure 6a,b,d,f). In contrast, the content of maslinic acid and ursolic acid from SD showed significant differences compared to other areas (Figure 6c,e). The maslinic acid content was 0.53 mg/cm^2^, four times higher than that of AH (0.13 mg/cm^2^), while the ursolic acid content from SD was 0.34 mg/cm^2^, three times higher than that of the lowest area, ZJ (0.11 mg/cm^2^). Thus, maslinic acid and ursolic acid can be used as SD-specific wax components for identifying fruit from the SD area.

**Figure 7:**
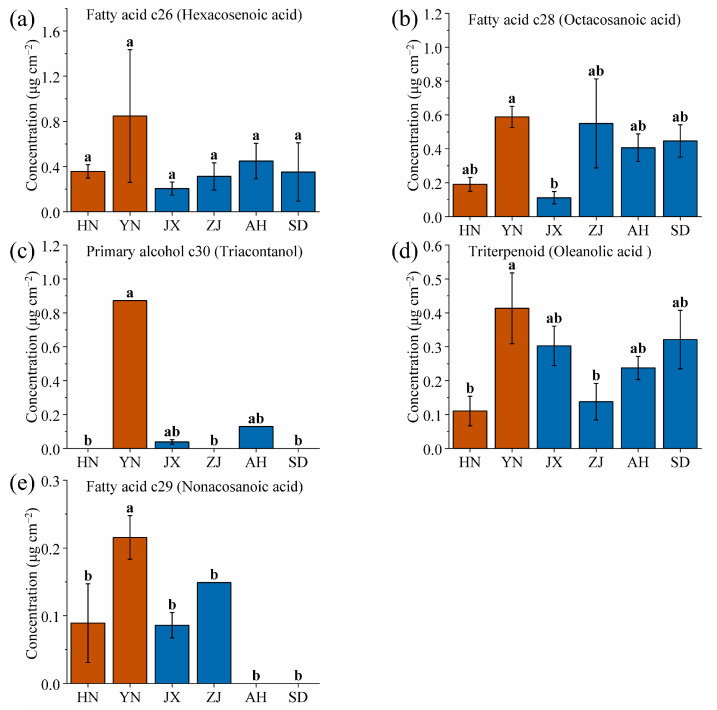
Comparison of (**a**) hexacosenoic acid, (**b**) octacosanoic acid, (**c**) triacontanol, (**d**) oleanolic acid, and (**e**) nonacosanoic acid wax contents of VF from six production areas (HN, YN, JX, ZJ, AH and SD). Different lowercase letters indicate significant differences, *p* < 0.05, Duncan’s HSD test.

Additionally, the five wax components with the highest content from YN were identified: hexacosenoic acid, octacosanoic acid, triacontanol, oleanolic acid, and nonacosanoic acid (Figure 7, significance analysis as shown in Table S3). Among these, three are fatty acids, suggesting that high-altitude environments favor the synthesis and accumulation of fatty acid waxes by VF. Among these components, the triacontanol content from YN showed a highly significant difference compared to the other five areas (Figure 7c). High-altitude environments are characterized by strong UV radiation and large diurnal temperature ranges. Triacontanol, with its long carbon chain, enhances the cuticular barrier’s ability to reflect UV radiation and reduce water loss, adapting to YN volcanic ash soil (which drains quickly, increasing drought stress) and lower atmospheric pressure (accelerating transpiration) [51,62]. The volcanic ash soil in YN (pH 5.5~6.8) provides optimal conditions for alcohol-forming enzymes, further promoting triacontanol synthesis. Specifically, the triacontanol content of VF from YN was 0.87 mg/cm^2^, a value that was undetectable in that from HN, ZJ, and SD, and only 0.04 and 0.13 mg/cm^2^ in that from JX and AH, respectively. Therefore, triacontanol can be used as a specific wax component for prioritizing the identification of the YN area.

Based on these findings, precise identification can be achieved between the YN and SD regions, which exhibit significant geographical differences. For VF from an unknown production area, the detection of triacontanol, maslinic acid, and ursolic acid waxes should be prioritized. If the triacontanol content exceeds 0.26 ± 0.05 μg/cm^2^, it can be identified as coming from the YN production area. Similarly, if the maslinic acid and ursolic acid contents exceed 0.53 ± 0.23 μg/cm^2^ and 0.26 ± 0.05 μg/cm^2^, respectively, the sample can be identified as coming from the SD production area.

### 2.3. Search for the Relationship Between VF Production Area and Latitude

**Figure 8:**
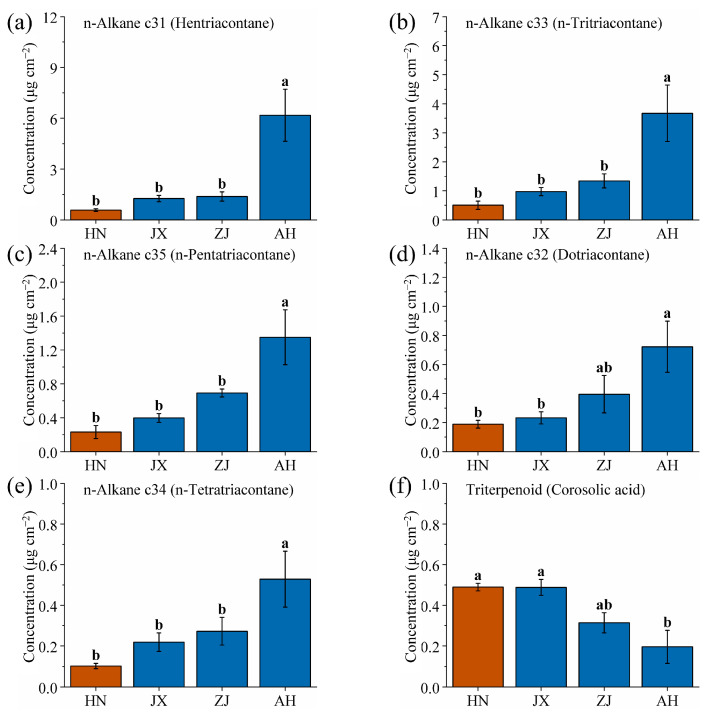
Comparison of (**a**) hentriacontane, (**b**) n-tritriacontane, (**c**) n-pentatriacontane, (**d**) dotriacontane, (**e**) n-tetratriacontane, and (**f**) corosolic acid wax contents of VF from four production areas (HN, JX, ZJ and AH). Different lowercase letters indicate significant differences, *p* < 0.05, Duncan’s HSD test.

After identifying the SD and YN production areas, this study plotted the n-alkane wax content of VF from the HN, JX, ZJ, and AH production areas in a column chart (Figure 8). The horizontal axis and color block representations are consistent with those of Figure 6. As the value on the horizontal axis increases, the content of n-alkane waxes also increases. Specifically, the content of hentriacontane, n-tritriacontane, n-pentatriacontane, dotriacontane, and n-tetratriacontane of VF increases monotonically with latitude across these production areas. HN (19.25° N) has a tropical climate (24~26 °C) and acidic laterite soil, leading to low n-alkane production—high temperatures accelerate transpiration, but excessive wax would trap heat, so plants minimize n-alkanes [44]. JX (27.12° N) and ZJ (28.97° N) have subtropical climates (17~19 °C, 1400~1600 mm precipitation) with red soil (pH 4.5~6.0). Moderate temperatures and rainfall here balance wax synthesis: n-alkanes increase to reduce water loss during seasonal dry periods but remain lower than in higher latitudes. AH (30.53° N), with a temperate climate (15~17 °C) and neutral soil, has the highest n-alkane content, as cooler temperatures reduce the risk of overheating, allowing plants to invest more in n-alkanes for winter water retention [63]. Additionally, the uniform increase in n-alkane wax content with latitude across different production areas may be related to the co-evolution of VF [64].

Furthermore, an inverse relationship was observed between the triterpenoid corosolic acid and the five n-alkane wax components (Figure 8f). The content of triterpenoid corosolic acid decreases with increasing latitude. This trend is likely due to a direct correlation between synthesis and conversion of n-alkanes and triterpenoids in the wax biosynthesis pathway of VF, where the synthesis of n-alkane waxes may inhibit synthesis of corosolic acid, or vice versa [65,66,67]. This trade-off reflects environmental adaptation: in high-latitude areas (e.g., AH), plants prioritize n-alkanes for physical protection (water retention, cold resistance) due to moderate precipitation, while in low-latitude areas (e.g., HN), higher corosolic acid levels combat oxidative stress from high humidity and UV radiation, as the need for chemical defense outweighs physical barrier reinforcement.

The monotonic correlation between the wax components (hentriacontane, n-tritriacontane, n-pentatriacontane, dotriacontane, n-tetratriacontane, and corosolic acid) of VF and the latitude of the production area is crucial for establishing a model that links wax content with latitude. This model can facilitate determination of the latitude and production area of VF by analyzing the wax content.

### 2.4. Establishment of a Model of the Relationship Between Single-Component Waxes and Latitude That Can Be Used to Identify the VF Production Area

**Figure 9:**
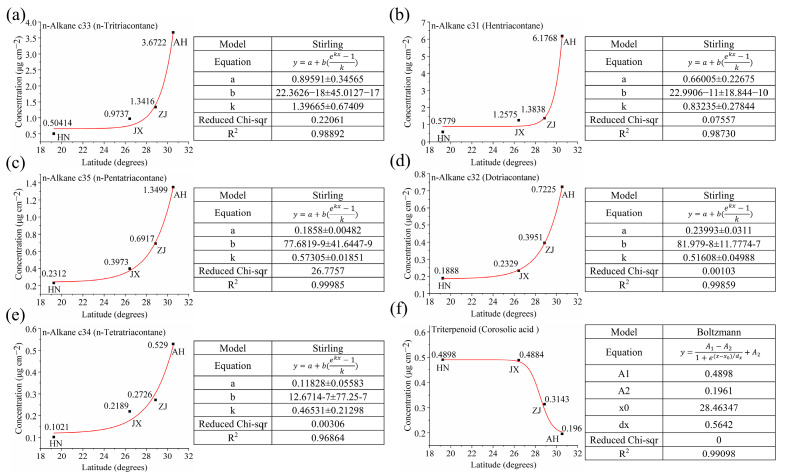
Fitting curves of (**a**) hentriacontane, (**b**) n-tritriacontane, (**c**) n-pentatriacontane, (**d**) dotriacontane, (**e**) n-tetratriacontane, and (**f**) corosolic acid waxes to latitude.

In this study, Origin 2024 was used to perform non-linear fitting of the six wax components (hentriacontane, n-tritriacontane, n-pentatriacontane, dotriacontane, n-tetratriacontane, and corosolic acid) against latitude (Figure 9).

Non-linear exponential models were selected over linear or polynomial fits based on the biological context of wax accumulation, which often follows saturation kinetics. Environmental factors act as “signals” to activate wax biosynthesis enzymes (e.g., fatty acid elongases), functioning analogously to the “substrate” component in saturation kinetics. When environmental stress at higher latitudes exceeds a threshold level, the activity of these enzymes stabilizes, resulting in diminished acceleration of wax accumulation and ultimately exhibiting characteristic saturation phenomena. Additionally, residual analysis confirmed that exponential models minimized heteroscedasticity and improved R^2^ values compared to linear alternatives.

The fitting process was iterated until the R^2^ value reached its maximum, or up to 1000 iterations, to ensure accuracy. The resulting regression equations for the six wax components are as follows:

(1) $$y_{1}=0.89591+(22.3626-18)({exp}^{1.39665x}-1)/1.39665$$

(2) $$y_{2}=0.66005+(22.9906-11)\left({exp}^{0.83235x}-1\right)/0.83235$$

(3) $$y_{4}=0.1858+(77.6819-9)\left({exp}^{0.57305x}-1\right)/0.57305$$

(4) $$y_{3}=0.23992+(81.979-8)\left({exp}^{0.51608x}-1\right)/0.51608$$

(5) $$y_{5}=0.11828+(12.6714-7)\left({exp}^{0.46531x}-1\right)/0.46531$$

(6) $$y_{6}=0.2937/\left(1+{exp}^{\left(x-28.46347\right)/0.5642}\right)+0.1961$$

*x* represents the latitude of the VF production area, and *y*_1_*_–_*_6_ represent the wax content of hentriacontane, n-tritriacontane, n-pentatriacontane, dotriacontane, n-tetratriacontane, and corosolic acid, respectively. The R^2^ values of regression Equations (1)~(6) are 0.98892, 0.98730, 0.99859, 0.99985, 0.96864, and 0.99098, respectively, indicating that the model accurately fits the data.

Furthermore, we transpose the model’s *x* and *y*, resulting in the following equations:

(7) $$y=0.716In(\left(1.22513-1.39665x_{1}\right)/22.3626)$$

(8) $$y=1.20142In(\left(0.54939-0.83235x_{2}\right)/22.9906)$$

(9) $$y=1.93768In(\left(0.12382-0.51608x_{3}\right)/81.979)$$

(10) $$y=1.74505In(\left(0.10647-0.57305x_{4}\right)/77.6819)$$

(11) $$y=2.1491In(\left(0.05503-0.46531x_{5}\right)/12.6714)$$

(12) $$y=0.5642In(1-0.2937/\left(x_{6}-0.1961\right))+28.46347$$

*x*_1_*_–_*_6_ represent the wax contents of hentriacontane, n-tritriacontane, n-pentatriacontane, dotriacontane, n-tetratriacontane, and corosolic acid to be determined for identification of the VF production area, while *y* represents the latitude of the VF production area.

For the VF from an unknown production area, if it is not identified as originating from SD or YN, the wax contents are analyzed individually according to models (7)~(12) [63,68,69]. By measuring any of the wax components (hentriacontane, n-tritriacontane, n-pentatriacontane, dotriacontane, n-tetratriacontane, or corosolic acid), the content of the wax component is substituted into the relevant model to predict the latitude of the production area. Combining these latitude data allows for accurate identification of the VF production area.

### 2.5. Establishment of a Model of the Relationship Between Multi-Component Waxes and Latitude That Can Be Used to Identify the VF Production Area

In addition, R Studio (4.4.1 version) was utilized to perform global fitting on the content of five wax components (hentriacontane, n-tritriacontane, n-pentatriacontane, dotriacontane, and n-tetratriacontane) to facilitate identification and verification of the VF production area based on multi-component wax profiles.

**Figure 10:**
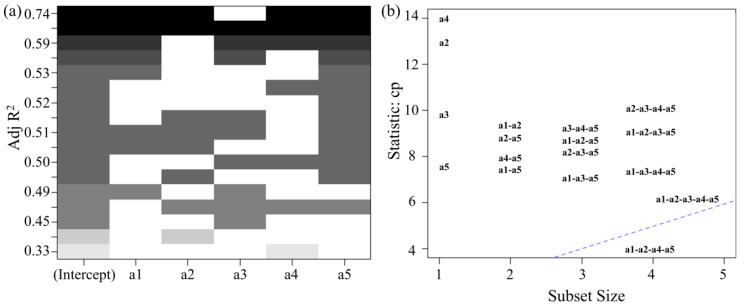
(**a**) Correlation heat-map of the content of five kinds of n-alkanes waxes with latitude. (**b**) PCA plot for all subsets of the five kinds of n-alkanes waxes.

The content of five wax components (hentriacontane, n-tritriacontane, n-pentatriacontane, dotriacontane, and n-tetratriacontane) was plotted in a scatter matrix, where a_1_ represents hentriacontane, a_2_ represents n-tritriacontane, a_3_ represents n-pentatriacontane, a_4_ represents dotriacontane, and a_5_ represents n-tetratriacontane. The data exhibit good normal distribution, and latitude positively correlates with wax content. After fitting the data, the final R^2^ value obtained was 0.6943, indicating relatively low model accuracy. Significance analysis identified a group of variables that negatively impacted prediction of the dependent variable, contributing to large fitting errors. To improve model accuracy, a heat-map was used to visualize the correlation between the data and latitude, helping to identify and exclude data groups that negatively affected the model (Figure 10a). The weakest correlation was observed between a_3_ and latitude, as indicated by the lightest color block. Additionally, PCA was performed on all subsets of the five wax components (Figure 10b). The PCA results showed that the combinations closest to the blue line (slope of 1) were a_1_-a_2_-a_4_-a_5_ and a_1_-a_2_-a_3_-a_4_-a_5_, further confirming that a_3_ differs significantly from the other data groups. This suggests that removing the a_3_ data would enhance the regression model’s accuracy.

After removing the a_3_ data, the a_1_, a_2_, a_4_, and a_5_ data were assessed. The final R^2^ obtained was 0.7375, indicating that removing the a_3_ data helps improve the accuracy of the regression model. Therefore, the regression model relating the wax content of VF to the latitude of the production area can be obtained.

(13) $$y=18.773-6.797*x_{1}+14.581*x_{2}-36.341*x_{4}+49.22*x_{5}$$

*x*_1_*_–_*_5_ represent the content of hentriacontane, n-tritriacontane, n-pentatriacontane, dotriacontane, and n-tetratriacontane, respectively, and *y* represents the latitude of the VF production area. According to this model, identification of the VF production area can be achieved using multi-component waxes.

For VF from unknown production areas, after identifying the specific production areas and confirming that the sample are not classified as being from SD or YN, multi-component wax analysis based on model (15) can be performed. The wax components (hentriacontane, n-tritriacontane, n-pentatriacontane, dotriacontane, and n-tetratriacontane) are detected from the unknown sample, and their contents are substituted into model (15) to estimate the latitude of the production area. By combining latitude data, the production area of the VF can be accurately determined.

### 2.6. Identification of the Production Area of VF Using the Multi-Component Wax Ratio

**Figure 11:**
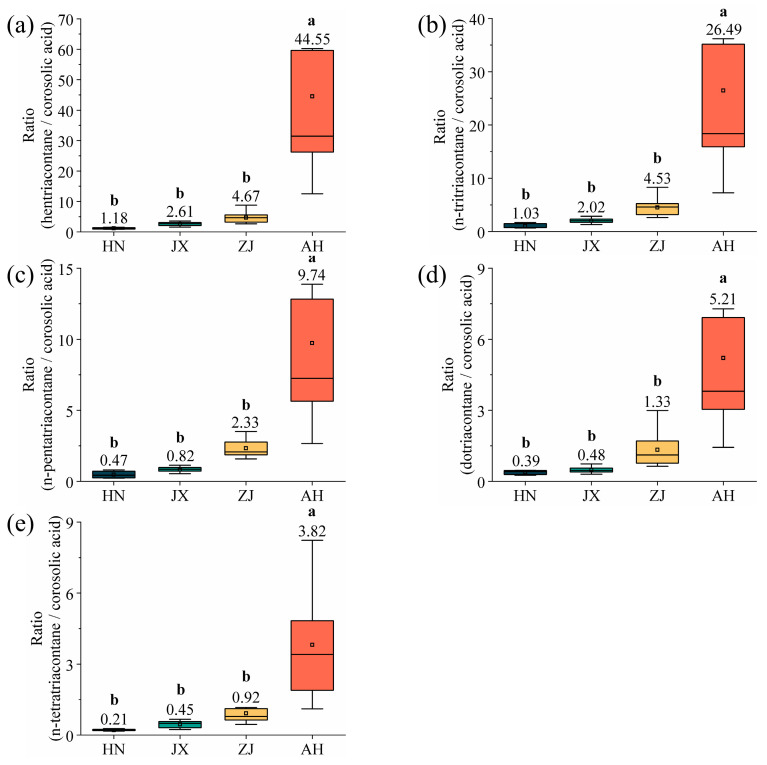
Wax ratio of (**a**) hentriacontane/corosolic acid, (**b**) n-tritriacontane/corosolic acid, (**c**) n-pentatriacontane/corosolic acid, (**d**) dotriacontane/corosolic acid, and (**e**) n-tetratriacontane/corosolic acid. Different lowercase letters indicate significant differences, *p* < 0.05, Duncan’s HSD test.

This study calculates the ratio of the wax components (hentriacontane, n-tritriacontane, n-pentatriacontane, dotriacontane, and n-tetratriacontane), whose contents increase monotonically with latitude, relative to corosolic acid, whose content decreases monotonically with latitude (Figure 11). This ratio is employed to more accurately determine the VF production area from multiple perspectives. The results reveal significant differences in the wax (hentriacontane, n-tritriacontane, n-pentatriacontane, dotriacontane, and n-tetratriacontane)-to-corosolic acid ratio among VF sample from various production areas, establishing it as an effective auxiliary method for production area identification. For VF from unknown production areas, i.e., not classified as SD or YN, the contents of hentriacontane, n-tritriacontane, n-pentatriacontane, dotriacontane, n-tetratriacontane, and corosolic acid can be detected to determine its production area. The formula is as follows:

(14) $$k_{1\text{–}5}=x_{1\text{–}5}/y$$

*x*_1_*_–_*_5_ represent the hentriacontane, n-tritriacontane, n-pentatriacontane, dotriacontane and n-tetratriacontane content, *y* represents the corosolic acid content, and *k*_1_*_–_*_5_ represent the ratio of multiple-component waxes.

The production area of VF can be determined based on the values of k_1_, k_2_, k_3_, k_4_, and k_5_. When k_1_ is 1.18 ± 0.06, k_2_ is 1.03 ± 0.05, k_3_ is 0.47 ± 0.02, k_4_ is 0.39 ± 0.02, and k_5_ is 0.21 ± 0.01, the production area is identified as HN. If k_1_ is 2.61 ± 0.13, k_2_ is 2.02 ± 0.10, k_3_ is 0.82 ± 0.04, k_4_ is 0.48 ± 0.02, and k_5_ is 0.45 ± 0.02, the production area is determined to be JX. When k_1_ is 4.67 ± 0.23, k_2_ is 4.53 ± 0.23, k_3_ is 2.33 ± 0.12, k_4_ is 1.33 ± 0.07, and k_5_ is 0.92 ± 0.05, the production area corresponds to ZJ. Lastly, when k_1_ is 44.55 ± 2.23, k_2_ is 26.49 ± 1.32, k_3_ is 9.74 ± 0.49, k_4_ is 5.21 ± 0.26, and k_5_ is 3.82 ± 0.19, the production area is identified as AH.

### 2.7. Investigate the Relationship Between Cuticular Waxes and VF Quality by Internal Metabolites

Using LC-MS, a total of 2786 metabolites were identified in the internal metabolism of VF. Based on previous research on the medicinal compounds of VF and other commonly used Chinese medicines, 10 classes of compounds with notable pharmacological effects and flavors were selected (phenols, quinolines and derivatives, flavonoids, isoflavonoids, coumarins and derivatives, morphinans, 3,4-dihydrocoumarins, organic acids, protopine alkaloids, and protoberberine alkaloids and derivatives), amounting to 93 components (Table S4). Previous studies have shown that VF from JX is of the highest quality among all production areas. Consequently, 19 metabolites from the JX production area were further screened from the 93, and they had significantly higher levels than those from other sources, marked as M_1_–M_19_ (highlighted in yellow in Table S4).

**Figure 12:**
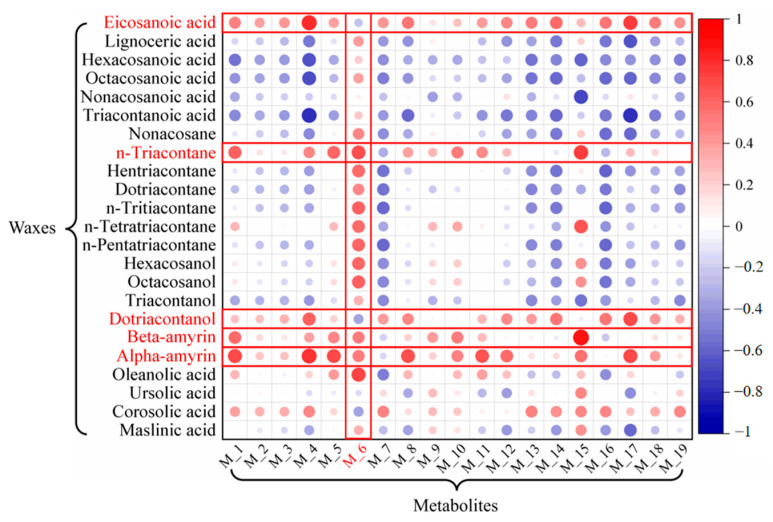
Correlation heat-map between metabolites and cuticular wax of VF.

A correlation heat-map analysis was conducted to assess the relationship between the high-content metabolites from the JX source and the wax components (Figure 12). The horizontal data indicate that the wax components—eicosanoic acid, n-triacontane, dotriacontanol, beta-amyrin, and alpha-amyrin—are represented by larger and darker circles, suggesting a strong positive correlation with the metabolites. Specifically, the higher the content of these wax components of VF, the higher the content of the primary medicinal compounds within the fruit.

Additionally, we combine the vertical data with five wax components—hentriacontane, n-tritriacontane, n-pentatriacontane, dotriacontane, and n-tetratriacontane—that show a monotonic correlation with latitude, and these components are highlighted in red (Figure 12). The results reveal that metabolite M_6_ (myricetin) exhibits the strongest positive correlation with these five wax components, which also show distinct latitude-dependent distribution patterns.

Further analysis reveals that the strong correlation between cuticular waxes and internal metabolites may arise from both shared metabolic precursors and the plant’s environmental adaptation needs. The significant positive correlation between n-triacontane and myricetin (M6) (Figure 12) is likely due to their common precursor, acetyl-CoA. In high-latitude areas, plants synthesize long-chain waxes by utilizing acetyl-CoA to enhance the fatty acid elongation pathway, while concurrently upregulating the phenylpropanoid pathway to promote flavonoid biosynthesis [70,71,72,73]. n-Triacontane reinforces the cuticular barrier to mitigate UV radiation damage, whereas myricetin acts as an intracellular antioxidant to scavenge reactive oxygen, forming a synergistic “external barrier—internal scavenging” mechanism to better adapt to high-latitude conditions characterized by intense UV radiation and winter cold stress [74,75]. In contrast, low-latitude plants exhibit lower cuticular wax content but higher flavonoid levels, likely due to preferential resource allocation to internal antioxidants for oxidative stress defense rather than extensive wax production in hot, humid climates. Meanwhile, soil pH also influences this balance, with neutral soils (typical of high-latitude areas) promoting triterpenoid-associated metabolites more efficiently than acidic soils (common in low-latitude areas) [76].

Moreover, the association of hentriacontane, n-tritriacontane, n-pentatriacontane, dotriacontane, and n-tetratriacontane with multiple bioactive metabolites suggests that these wax components may function as signaling molecules to activate downstream metabolic pathways. Previous studies have demonstrated that triterpenoids can coordinately regulate both wax biosynthesis and flavonoid accumulation through modulation of the MYB transcription factor family [77,78]. This coordinated mechanism likely represents an adaptive strategy developed by *Vitex trifolia* L. and *Vitex rotundifolia* L. f. during long-term geographic expansion, whereby synergistic adjustments in cuticular waxes and internal metabolites optimize adaptation efficiency across diverse climatic zones.

## 3. Materials and Methods

### 3.1. Reagents

Chloroform was purchased from General-Reagent (Shanghai, China). Pyridine, tetracosane, anhydrous sodium sulfate, N,O-Bis (trimethylsilyl) trifluoroacetamide (BSTFA), and 2-chloro-L-phenylalanine were obtained from Aladdin (Shanghai, China). Acetonitrile and methanol were sourced from Thermo (Loughborough, UK), while formic acid was supplied by TCL (Shanghai, China), and ammonium formate was supplied by Sigma (Shanghai, China). Nitrogen gas was procured from Liufang Industrial Gas Co., Ltd. (Suzhou, China).

### 3.2. Selection of VF Sample for Cuticular Wax Extraction

**Figure 13:**
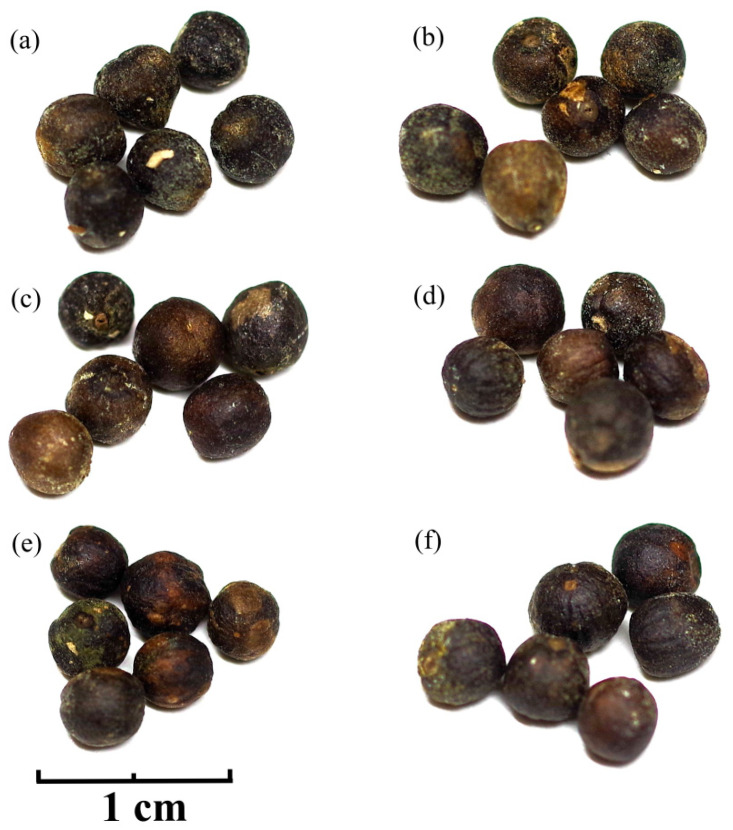
Optical photos of VF from various production areas, (**a**) HN, (**b**) YN, (**c**) JX, (**d**) ZJ, (**e**) AH, and (**f**) SD.

VF was harvested from six major production areas in China: Qionghai City, Hainan Province (from *Vitex trifolia* L. in mid-June 2024), Tengchong City, Yunnan Province (from *Vitex trifolia* L. in early July 2024), Ji’an City, Jiangxi Province (from *Vitex rotundifolia* L. f. in mid-July 2024), Quzhou City, Zhejiang Province (from *Vitex rotundifolia* L. f. in late July 2024), Anqing City, Anhui Province (from *Vitex rotundifolia* L. f. in early August 2024), and Yantai City, Shandong Province (from *Vitex rotundifolia* L. f. in mid-August 2024). The plants from these production areas had been growing in stable environments for several years, yielding medium-sized VF. Sample from these areas were labeled as HN, YN, JX, ZJ, AH, and SD, respectively (Figure 13).

Soil and climate indicators of the different sampling sites as shown in Table 2. The latitude and altitude data were sourced from Google Earth (https://earth.google.com, accessed on 5 July 2025). The soil type and soil pH data were obtained from the Institute of Soil Science, Chinese Academy of Sciences (http://issas.cas.cn, accessed on 5 July 2025) and World Soil Information (ISRIC)—SoilGrids (https://soilgrids.org, accessed on 5 July 2025). The climate data were derived from China Meteorological Data Network (http://data.cma.cn, accessed on 6 July 2025) and National Climate Center (http://cmdp.ncc-cma.net, accessed on 6 July 2025).

After harvesting, the VF sample were dried at 45 °C and stored in a cool environment. Each batch was divided into three subgroups, with six undamaged fruits selected from each subgroup for cuticular wax extraction. The diameter of each VF was measured using a caliper (both transverse and longitudinal diameters were recorded three times each) to calculate the cuticular area based on a spherical model.

For wax extraction, each subgroup was immersed in 6 mL of chloroform for 2 min. Anhydrous sodium sulfate (1 g) was added to the extract, and impurities were removed using neutral filter paper. The extract was then dried with nitrogen gas to obtain the dried wax. The dried wax was dissolved and derivatized in pyridine and N,O-Bis (trimethylsilyl) trifluoroacetamide (BSTFA) at 70 °C for 30 min. Finally, the sample was diluted with chloroform and analyzed using GC-MS [79].

### 3.3. GC-MS Detection of Wax Extracts

GC-MS analysis was performed using a gas chromatograph equipped with a mass spectrometric detector (*m*/*z* 50–750, MSD 5975; Agilent Technologies, Santa Clara, USA) and a capillary column (30 m × 0.32 mm, DB-1 ms, 0.1 μm film; J&W Scientific, Agilent Technologies, Santa Clara, USA). A 1 μL aliquot of each sample was injected into the system. The chromatographic temperature program was set as follows: an initial temperature of 50 °C, held for 2 min, followed by a ramp of 40 °C/min to 200 °C, held for 2 min, increased at 3 °C/min to 320 °C, and maintained at 320 °C for 30 min [80,81,82].

Retention times were compared and evaluated using the Wiley 10th/National Institute of Standards and Technology (NIST) 2017 Mass Spectral Library and LipidWeb data (https://www.lipidhome.co.uk, accessed on 12 October 2024). Peak intensity signals were segmented and normalized based on internal standards, with an RSD > 0.3. After normalization, redundant signals were removed, and peak merging was performed to generate a refined data matrix [83]. The obtained GC-MS data were then used to determine the wax content. This information, combined with the measured cuticular area, was utilized to calculate the wax content per unit cuticular area for wax scales from different production areas. The formula is as follows:

(15) $$s=4\Pi r^{2}$$

(16) $$c=m/\left(s_{1}+s_{2}+s_{3}+s_{4}+s_{5}+s_{6}\right)$$

where *r* represents the average value of the transverse and longitudinal diameter of the VF (cm), *s* represents the cuticular area of the VF (cm^2^), *s*_1_*_–_*_6_ represent the cuticular areas of six VF in each group (cm^2^), *m* represents the mass of wax components in different groups (μg), and *c* represents the wax concentration per unit cuticular area of the VF—that is, the wax content on the cuticle of the VF (μg/cm^2^).

### 3.4. Internal Metabolomics LC-MS Detection of VF

Quantitative VF sample were placed in a 2 mL centrifuge tube, followed by the addition of 600 µL of methanol and 2-chloro-L-phenylalanine (4 ppm). The mixture was stirred thoroughly to ensure proper pre-treatment. The pre-treated sample was then transferred to a tissue grinder and homogenized for 1 min at a frequency of 55 Hz. After resting at room temperature for 15 min, the sample was centrifuged at 12,000 rpm for 10 min. The resulting supernatant was filtered through a 0.22 μm membrane to obtain the final liquid sample for analysis. The prepared sample was subsequently analyzed using liquid chromatography–mass spectrometry (LC-MS, Thermo Fisher Scientific, Waltham, MA, USA) [84,85].

LC analysis was performed using a Vanquish UHPLC System (Chromeleon, version: 7.3.2, Thermo Fisher Scientific, Waltham, USA). Chromatographic separation was conducted on an ACQUITY UPLC^®^ HSS T3 column (2.1 × 100 mm, 1.8 µm; Waters, Milford, MA, USA) maintained at 40 °C. The flow rate was set at 0.3 mL/min, with an injection volume of 2 μL.

For LC-ESI (+)-MS analysis, the mobile phase consisted of (B2) 0.1% formic acid in acetonitrile (*v*/*v*) and (A2) 0.1% formic acid in water (*v*/*v*). The gradient program was as follows: 0~1 min, 8% B2; 1~8 min, 8~98% B2; 8~10 min, 98% B2; 1~10.1 min, 98~8% B2; 10.1~12 min, 8% B2.

For LC-ESI (−)-MS analysis, the mobile phase consisted of (B3) acetonitrile and (A3) 5 mM ammonium formate. The gradient conditions were as follows: 0~1 min, 8% B3; 1~8 min, 8~98% B3; 8~10 min, 98% B3; 10~10.1 min, 98~8% B3; 10.1~12 min, 8% B3 [86].

Metabolite detection was performed using an Orbitrap Exploris 120 mass spectrometer (Thermo Fisher Scientific, Waltham, USA) equipped with an electrospray ionization (ESI) source. Simultaneous MS1 and MS/MS data acquisition was conducted in Full MS-ddMS2 mode (data-dependent MS/MS). The parameters were as follows: sheath gas pressure: 40 arb; auxiliary gas flow: 10 arb; spray voltage: 3.50 kV (ESI+) and −2.50 kV (ESI−); capillary temperature: 325 °C; MS1 scan range: *m*/*z* 100~1000; MS1 resolving power: 60,000 FWHM; number of data-dependent scans per cycle: 4; MS/MS resolving power: 15,000 FWHM; normalized collision energy: 30%; dynamic exclusion time: automatic [87].

### 3.5. Statistical Analysis

Three biological replicates were conducted for each group, and the results were expressed as the mean and standard deviation. Group differences were analyzed by one-way analysis of variance (ANOVA) followed by Duncan’s HSD post hoc test (*p* < 0.05) to control for multiple comparisons, ensuring robust identification of latitude-dependent wax variations.

For wax composition analysis, single-component wax nonlinear regression modeling was conducted using Origin 2024. Multi-component wax analysis involved a scatter matrix plot, principal component analysis (PCA) through subset regression, and linear regression modeling, all performed using R Studio (4.4.1 version).

## 4. Conclusions

This study establishes that VF cuticular waxes serve as robust chemotaxonomic markers for both geographical authentication and quality prediction. The pronounced latitudinal variations in wax composition reflect adaptive responses to climatic stressors, with high-latitude regions favoring waterproofing n-alkanes and high-altitude sites synthesizing UV-protective triacontanol. Crucially, the eicosanoic acid, n-triacontane, dotriacontanol, alpha/beta-amyrin wax components correlated strongly with internal bioactive metabolites, validating waxes as dual biomarkers for production area and pharmacological potency.

Our developed authentication models—single-component, multi-component, and wax ratio methods—achieved high accuracy in discriminating production areas. Mechanistically, the inverse relationship between n-alkanes and corosolic acid suggests a trade-off in carbon allocation between physical barrier formation and oxidative stress defense. This adaptive strategy aligns with ecological gradients, where low-latitude plants prioritize chemical defenses, while temperate cultivars invest in cuticular waterproofing.

This study resolves the persistent challenge of VF production area authentication by demonstrating that cuticular waxes serve as stable “environmental adaptation fingerprints,” overcoming the limitations of traditional metabolite-based methods. The developed biomarkers and protocols provide a transformative framework for geographical authentication and quality assessment of medicinal plants, enabling more precise agriculture and standardized herbal product development.

## Figures and Tables

**Table 1:** Wax constituents identified in VF extracts, their retention times (min), and their relative contents from each production area sample (HN, YN, JX, ZJ, AH, and SD). Note: The lowercase letters following percent value indicate the significance of its difference compared to other production areas. *p* < 0.05, Duncan’s HSD test.

Retention Times (min)	Waxes	HN			YN			JX			ZJ			AH			SD		
		(%)	SD	CV	(%)	SD	CV	(%)	SD	CV	(%)	SD	CV	(%)	SD	CV	(%)	SD	CV
14.738	Fatty acid c24 (Lignoceric acid)	8.017 ^ab^	0.005	0.067	6.190 ^abc^	0.013	0.214	4.722 ^bc^	0.019	0.392	4.351 ^c^	0.018	0.409	6.162 ^abc^	0.006	0.102	8.688 ^a^	0.010	0.118
15.664	n-Alkane c29 (Nonacosane)	7.816 ^c^	0.004	0.057	11.119 ^b^	0.017	0.154	6.754 ^c^	0.018	0.263	4.680 ^c^	0.021	0.439	14.636 ^ab^	0.009	0.062	15.323 ^a^	0.010	0.067
16.522	Primary alcohol c26 (Hexacosanol)	2.439 ^a^	0.014	0.592	3.119 ^a^	0.007	0.212	3.404 ^a^	0.016	0.473	3.260 ^a^	0.009	0.271	1.592 ^a^	0.006	0.372	4.202 ^a^	0.013	0.316
17.108	n-Alkane c30 (n-Triacontane)	10.905 ^bc^	0.021	0.195	8.086 ^bc^	0.033	0.409	14.381 ^ab^	0.022	0.153	18.009 ^a^	0.071	0.394	4.248 ^c^	0.009	0.216	8.850 ^bc^	0.050	0.571
17.805	Fatty acid c26 (Hexacosenoic acid)	5.855 ^a^	0.017	0.296	5.534 ^a^	0.035	0.628	2.258 ^a^	0.004	0.196	3.041 ^a^	0.027	0.877	2.150 ^a^	0.007	0.303	2.133 ^a^	0.013	0.623
18.680	n-Alkane c31 (Hentriacontane)	9.482 ^d^	0.012	0.131	22.007 ^b^	0.009	0.041	13.852 ^c^	0.025	0.180	13.451 ^c^	0.008	0.057	29.579 ^a^	0.013	0.043	20.125 ^b^	0.011	0.052
19.623	Primary alcohol c28 (Octacosanol)	1.719 ^a^	0.002	0.129	1.444 ^a^	0.002	0.120	1.791 ^a^	0.002	0.121	2.166 ^a^	0.008	0.355	1.515 ^a^	0.010	0.635	1.711 ^a^	0.018	1.068
20.243	n-Alkane c32 (Dotriacontane)	3.099 ^a^	0.003	0.091	3.154 ^a^	0.003	0.102	2.565 ^ab^	0.002	0.065	3.841 ^a^	0.004	0.111	3.460 ^a^	0.003	0.090	1.684 ^b^	0.007	0.393
20.421	Beta-amyrin	1.818 ^b^	0.002	0.094	1.461 ^b^	0.003	0.218	3.635 ^b^	0.005	0.147	2.625 ^ab^	0.009	0.341	2.052 ^b^	0.004	0.202	3.014 ^ab^	0.009	0.288
20.931	Alpha-amyrin	2.172 ^b^	0.001	0.052	2.185 ^b^	0.007	0.299	4.432 ^a^	0.012	0.273	2.593 ^b^	0.009	0.365	2.274 ^b^	0.003	0.152	1.785 ^b^	0.009	0.531
21.093	Fatty acid c28 (Octacosanoic acid)	3.125 ^ab^	0.005	0.144	3.844 ^a^	0.009	0.221	1.226 ^b^	0.002	0.136	5.344 ^a^	0.017	0.313	1.298 ^b^	0.009	0.710	2.703 ^ab^	0.010	0.367
21.959	n-Alkane c33 (n-Tritiacontane)	8.272 ^d^	0.017	0.209	14.553 ^b^	0.010	0.066	10.726 ^c^	0.013	0.122	13.041 ^bc^	0.010	0.079	17.585 ^a^	0.010	0.060	11.216 ^c^	0.007	0.066
22.630	Fatty acid c29 (Nonacosanoic acid)	1.463 ^a^	0.007	0.475	1.407 ^a^	0.006	0.436	0.946 ^a^	0.001	0.047	1.448 ^a^	0.005	0.319	0 ^a^	0	—	0 ^a^	0	—
22.936	Primary alcohol c30 (Triacontanol)	0 ^a^	0	—	5.695 ^a^	0.027	0.466	0.430 ^a^	0.001	0.167	0 ^a^	0	—	0.620 ^a^	0.004	0.663	0 ^a^	0	—
23.395	n-Alkane c34 (n-Tetratriacontane)	1.676 ^a^	0.003	0.160	1.530 ^a^	0.009	0.558	2.412 ^a^	0.004	0.183	2.651 ^a^	0.002	0.082	2.533 ^a^	0.012	0.462	2.496 ^a^	0.008	0.318
23.726	Fatty acid c30 (Triacontanoic acid)	2.987 ^a^	0.001	0.032	1.831 ^a^	0.009	0.466	1.038 ^a^	0.007	0.672	1.206 ^a^	0.005	0.453	0.996 ^a^	0.007	0.663	1.835 ^a^	0.009	0.517
24.525	Oleanolic acid	1.812 ^ab^	0.008	0.434	2.700 ^ab^	0.009	0.316	3.334 ^a^	0.005	0.141	1.340 ^ab^	0.010	0.745	1.138 ^b^	0.004	0.390	1.945 ^ab^	0.010	0.531
25.213	Ursolic acid	4.262 ^a^	0.004	0.091	0.842 ^d^	0.003	0.359	2.317 ^b^	0.002	0.106	1.090 ^cd^	0.004	0.331	0.783 ^d^	0.003	0.363	2.042 ^bc^	0.008	0.389
25.493	n-Alkane c35 (n-Pentatriacontane)	3.795 ^b^	0.011	0.287	5.679 ^ab^	0.021	0.372	4.377 ^ab^	0.006	0.146	6.724 ^a^	0.016	0.231	6.464 ^ab^	0.005	0.081	3.731 ^ab^	0.015	0.401
27.226	Primary alcohol c32 (Dotriacontanol)	2.351 ^a^	0.006	0.252	0.424 ^b^	0.002	0.406	1.859 ^a^	0.003	0.154	1.774 ^a^	0.003	0.176	0 ^b^	0	—	0 ^b^	0	—
28.033	Maslinic acid	4.282 ^a^	0.007	0.152	1.967 ^bc^	0.011	0.539	2.856 ^ab^	0.005	0.166	2.648 ^abc^	0.005	0.206	0.620 ^c^	0.002	0.256	3.218 ^ab^	0.012	0.359
28.356	Corosolic acid	8.036 ^a^	0.006	0.077	1.157 ^d^	0.005	0.389	5.380 ^b^	0.010	0.190	3.055 ^c^	0.015	0.489	0.939 ^d^	0.003	0.268	2.694 ^cd^	0.011	0.417

“—” indicates that the data were not detected.

**Table 2:** Soil and climate indicators (environmental factors) of the different sampling sites.

Production Areas	Average Latitude	Average Altitude (m)	Soil Types	Soil pH	Annual Average Temperature (°C)	Annual Average Precipitation (mm)	Frost-Free Period (days)
Qionghai City Hainan Province	19.25° N	10~50	Laterite soil Red soil	5.0~6.5	24~26	2000~2500	365
Tengchong City Yunnan Province	25.02° N	1600~1900	Red soil Volcanic ash soil	5.5~6.8	14~16	1400~1600	270~300
Ji’an City Jiangxi Province	27.12° N	50~200	Red soil Paddy soil	4.5~6.0	17~19	1400~1600	280~300
Quzhou City Zhejiang Province	28.97° N	50~150	Red soil Yellow soil	5.0~6.5	16~18	1500~1800	250~280
Anqing City Anhui Province	30.53° N	20~100	Yellow-brown soil Paddy soil	5.5~7.0	15~17	1300~1500	240~260
Yantai City Shandong Province	37.52° N	10~100	Brown soil Brown soil	6.5~7.5	11~13	550~700	200~220

**Table 3:** The contents and percentages of each primary wax class (fatty acid, n-alkane, primary alcohol, and triterpenoid) from each production area sample (HN, YN, JX, ZJ, AH, and SD). Note: The lowercase letters following content mean value indicate the significance of its difference compared to other production areas. *p* < 0.05, Duncan’s HSD test.

	HN			YN			JX			ZJ			AH			SD		
Content	Mean	SD	(%)	Mean	SD	(%)	Mean	SD	(%)	Mean	SD	(%)	Mean	SD	(%)	Mean	SD	(%)
μg/cm^2^																		
Total fatty acids	1.59 ^a^	0.25	26.06	2.60 ^a^	1.04	16.96	1.41 ^a^	0.64	15.49	1.75 ^a^	1.00	17.05	2.45 ^a^	1.01	11.72	2.64 ^a^	1.16	15.96
Total n-alkanes	2.75 ^a^	0.50	45.04	10.13 ^abc^	2.38	66.13	5.00 ^bc^	1.55	55.07	6.42 ^bc^	1.75	62.40	16.22 ^a^	7.00	77.66	10.47 ^ab^	5.56	63.43
Total primary alcohols	0.40 ^a^	0.18	6.51	1.01 ^a^	0.48	6.60	0.68 ^a^	0.39	7.48	0.74 ^a^	0.29	7.20	0.59 ^a^	0.44	2.81	0.98 ^a^	0.35	5.91
Total triterpenoids	1.36 ^a^	0.17	22.38	1.58 ^a^	0.42	10.31	1.99 ^a^	0.67	21.96	1.37 ^a^	0.47	13.35	1.63 ^a^	0.64	7.81	2.43 ^a^	1.39	14.70
Total	6.09 ^b^			15.31 ^ab^			9.08 ^b^			10.29 ^b^			20.88 ^a^			16.51 ^ab^		

**Table 4:** All wax component contents from each production area sample (HN, YN, JX, ZJ, AH, and SD). Note: The lowercase letters following content mean value indicate the significance of its difference compared to other production areas. *p* < 0.05, Duncan’s HSD test.

Classification	Content	HN			YN			JX			ZJ			AH			SD		
	μg/cm^2^	Mean	SD	(%)	Mean	SD	(%)	Mean	SD	(%)	Mean	SD	(%)	Mean	SD	(%)	Mean	SD	(%)
Fatty	Tetracosanoic acid	0.37 ^a^	0.20	6.01	0.16 ^a^	0.08	1.01	0.51 ^a^	0.53	5.63	0.27 ^a^	0.13	2.60	0.37 ^a^	0.17	1.74	0.20 ^a^	0.08	1.21
	Lignoceric acid	0.49 ^b^	0.13	7.90	0.95 ^ab^	0.20	5.78	0.43 ^b^	0.05	4.71	0.45 ^b^	0.43	4.31	1.29 ^a^	0.49	6.02	1.43 ^a^	0.49	8.64
	Hexacosenoic acid	0.36 ^a^	0.10	5.77	0.85 ^a^	0.83	5.17	0.21 ^a^	0.10	2.25	0.31 ^a^	0.21	3.01	0.45 ^a^	0.27	2.10	0.35 ^a^	0.45	2.12
acids	Octacosanoic acid	0.19 ^ab^	0.07	3.08	0.59 ^a^	0.11	3.59	0.11 ^b^	0.06	1.22	0.55 ^ab^	0.46	5.29	0.27 ^ab^	0.25	1.27	0.45 ^ab^	0.17	2.69
	Nonacosanoic acid	0.09 ^b^	0.08	1.44	0.22 ^a^	0.05	1.32	0.09 ^b^	0.03	0.94	0.15 ^b^	—	1.43	0 ^b^	—	0	0 ^b^	—	0
	Triacontanoic acid	0.18 ^a^	0.05	2.94	0.28 ^a^	0.13	1.71	0.09 ^a^	0.06	1.03	0.12 ^a^	0.13	1.19	0.21 ^a^	—	0.97	0.30 ^a^	0.14	1.82
n-Alkanes	Nonacosane	0.48 ^b^	0.08	7.70	1.70 ^ab^	0.29	10.39	0.61 ^b^	0.24	6.73	0.48 ^b^	0.39	4.64	3.06 ^a^	1.38	14.29	2.53 ^a^	1.21	15.23
	n-Triacontane	0.66 ^a^	0.09	10.75	1.24 ^a^	0.81	7.56	1.31 ^a^	0.67	14.33	1.85 ^a^	0.42	17.84	0.89 ^a^	0.18	4.15	1.46 ^a^	1.31	8.80
	Hentriacontane	0.58 ^c^	0.14	9.34	3.37 ^b^	0.57	20.57	1.26 ^bc^	0.33	13.80	1.38 ^bc^	0.47	13.32	6.18 ^a^	2.66	28.88	3.32 ^b^	1.50	20.00
	Dotriacontane	0.19 ^b^	0.05	3.05	0.48 ^ab^	0.04	2.95	0.23 ^b^	0.07	2.56	0.40 ^b^	0.22	3.80	0.72 ^a^	0.31	3.38	0.28 ^b^	0.16	1.67
	n-Tritriacontane	0.50 ^c^	0.25	8.15	2.23 ^ab^	0.45	13.60	0.97 ^bc^	0.24	10.69	1.34 ^bc^	0.42	12.92	3.67 ^a^	1.68	17.17	1.85 ^bc^	0.96	11.15
	n-Tetratriacontane	0.10 ^b^	0.02	1.65	0.23 ^ab^	0.16	1.43	0.22 ^ab^	0.08	2.40	0.27 ^ab^	0.12	2.63	0.53 ^a^	0.19	2.47	0.41 ^ab^	0.34	2.48
	n-Pentatriacontane	0.23 ^c^	0.13	3.74	0.87 ^ab^	0.50	5.31	0.40 ^bc^	0.09	4.36	0.69 ^bc^	0.08	6.66	1.35 ^a^	0.56	6.31	0.62 ^bc^	0.21	3.71
Primary	Hexacosanol	0.15 ^a^	0.14	2.40	0.48 ^a^	0.07	2.92	0.31 ^a^	0.29	3.39	0.34 ^a^	0.26	3.23	0.33 ^a^	0.13	1.55	0.69 ^a^	0.60	4.18
	Octacosanol	0.10 ^a^	0.04	1.69	0.22 ^a^	0.04	1.35	0.16 ^a^	0.04	1.78	0.22 ^a^	0.01	2.14	0.32 ^a^	0.45	1.48	0.28 ^a^	0.25	1.70
alcohols	Triacontanol	0 ^b^	—	0	0.87 ^a^	—	5.32	0.04 ^ab^	0.02	0.43	0 ^b^	—	0	0.13 ^ab^	—	0.60	0 ^b^	—	0
	Dotriacontanol	0.14 ^a^	0.05	2.32	0.06 ^b^	—	0.40	0.17 ^a^	0.06	1.85	0.18 ^a^	0.04	1.76	0 ^b^	—	0	0 ^b^	—	0
Triterpenoids	Beta-amyrin	0.11 ^a^	0.03	1.79	0.22 ^a^	0.07	1.37	0.33 ^a^	0.18	3.62	0.27 ^a^	0.19	2.60	0.43 ^a^	0.28	2.00	0.50 ^a^	0.38	3.00
	Alpha-amyrin	0.13 ^b^	0.03	2.14	0.33 ^ab^	0.12	2.04	0.40 ^a^	0.21	4.42	0.27 ^ab^	0.16	2.57	0.47 ^a^	0.13	2.22	0.29 ^ab^	0.03	1.77
	Oleanolic acid	0.11 ^b^	0.08	1.79	0.41 ^a^	0.18	2.52	0.30 ^ab^	0.10	3.32	0.14 ^b^	0.09	1.33	0.24 ^ab^	0.06	1.11	0.32 ^ab^	0.15	1.93
	Ursolic acid	0.26 ^ab^	0.08	4.20	0.13 ^ab^	0.05	0.79	0.21 ^ab^	0.10	2.31	0.11 ^b^	0.02	1.08	0.16 ^ab^	0.03	0.76	0.34 ^a^	0.23	2.03
	Corosolic acid	0.49 ^a^	0.03	7.92	0.18 ^b^	0.09	1.08	0.49 ^a^	0.07	5.36	0.31 ^ab^	0.09	3.03	0.20 ^b^	0.14	0.92	0.44 ^ab^	0.30	2.68
	Maslinic acid	0.26 ^ab^	0.05	4.22	0.30 ^ab^	0.20	1.84	0.26 ^ab^	0.06	2.85	0.27 ^ab^	0.18	2.62	0.13 ^b^	0.07	0.60	0.53 ^a^	0.39	3.20
	Total	6.18 ^b^	—	—	16.39 ^ab^	—	—	9.11 ^b^	—	—	10.39 ^b^	—	—	21.39 ^a^	—	—	16.61 ^ab^	—	—

“—” indicates that the data were not detected.

## Data Availability

The data presented in this study are available on request from the corresponding author due to privacy.

## Supplementary Materials

The following supporting information can be downloaded at: https://www.mdpi.com/article/10.3390/ijms26157293/s1**.**
