# Supplementary material for: Preliminary Study on the Geochemical Characterization of Viticis Fructus Cuticular Waxes: From Latitudinal Variation to Origin Authentication
Source: Int J Mol Sci. 2025 Jul 28;26(15):7293. doi: 10.3390/ijms26157293 (PMC12347355; doi:10.3390/ijms26157293)
Supplement: Supplementary file 1 [file ijms-26-07293-s001.zip › ijms-3732693-supplementary.pdf]

Supplementary Material

for

Preliminary Study on Geochemical characterization of Viticis Fructus Cuticular Waxes: From Latitudinal Variation to Origin Authentication

†Yiqing Luo[1 2 3], †Min Guo[2 3], Lei Hu[2 3], Jiaxin Yang[2 3], Junyu Xu[2 4] Muhammad Rafiq[2], Ying Wang[1], Chunsong Cheng[2 3 *], Shaohua Zeng[1 *]

[1] *Guangdong Provincial Key Laboratory of Applied Botany, South China Botanical Garden, Chinese Academy of Sciences, Guangzhou City, 510650, PR China*

[2] *Jiangxi Key Laboratory for Sustainable Utilization of Chinese Materia Medica Resources, Lushan Botanical Garden, Chinese Academy of Sciences, Jiujiang City, 332900, PR China*

[3] *Lushan Xinglin Institute for Medicinal Plants & Jiujiang Xinglin Key Laboratory for Traditional Chinese Medicines, Lushan, Jiujiang City, 332900, PR China*

[4] *School of Life Sciences, Nanchang University, Nanchang City, 330031, PR China*

* Corresponding author:
chengcs@lsbg.com (C. Cheng)
shhzeng@scbg.ac.cn (Sh. Zeng)

Table S1 The significance analysis of n-alkanes c31, c33, c35, c32, and c34 waxes (Subset of alpha = 0.05. PA is production area. Duncan [a] and Duncan [b] represent harmonic mean sample size = 3.00 and 2.769, respectively).

		PA	N	c	b	a
n-Alkanes c31	Duncan [a]	HN	3	0.5778667		
		JX	3	1.2575333	1.2575333	
		ZJ	3	1.3838333	1.3838333	
		SD	3		3.3221667	
		YN	3		3.3700667	
		AH	3			6.1768667
	Significance			0.482	0.088	1
		PA	N	c	b	a
n-Alkanes c33	Duncan [a]	HN	3	0.5041333		
		JX	3	0.9737333	0.9737333	
		ZJ	3	1.3416333	1.3416333	
		SD	3	1.8515667	1.8515667	
		YN	3		2.2286333	2.2286333
		AH	3			3.6722333
	Significance			0.093	0.116	0.057
		PA	N	c	b	a
n-Alkanes c35	Duncan [a]	HN	3	0.2312667		
		JX	3	0.3973333	0.3973333	
		SD	3	0.6158667	0.6158667	
		ZJ	3	0.6917	0.6917	
		YN	3		0.8697	0.8697
		AH	3			1.3499667
	Significance			0.135	0.126	0.097
		PA	N	b	a	
n-Alkanes c32	Duncan [a]	HN	3	0.1888333		
		JX	3	0.2329		
		SD	3	0.2779667		
		ZJ	3	0.3951667		
		YN	3	0.4829333	0.4829333	
		AH	3		0.7225667	
	Significance			0.079	0.113	
		PA	N	b	a	
n-Alkanes c34	Duncan [b]	HN	3	0.1021333		
		JX	3	0.2189667	0.2189667	
		YN	3	0.2343333	0.2343333	
		ZJ	3	0.2727	0.2727	
		SD	3	0.4121	0.4121	
		AH	2		0.529	
	Significance			0.094	0.094	

Table. S2 The significance analysis of fatty acids c24, primary alcohols c26, maslinic acid, beta-amyrin, ursolic acid, and fatty acids c30 waxes (Subset of alpha = 0.05. PA is production area. Duncan [a] and Duncan [c] represent harmonic mean sample size = 3.00 and 2.400, respectively).

		PA	N	b	a
Fatty acids c24	Duncan [a]	JX	3	0.4287	
		ZJ	3	0.4476333	
		HN	3	0.4886	
		YN	3	0.9479	0.9479
		AH	3		1.2868667
		SD	3		1.4342667
	Significance			0.116	0.129
		PA	N	a	
Primary alcohols c26	Duncan [a]	HN	3	0.1486333	
		JX	3	0.3090667	
		AH	3	0.3325	
		ZJ	3	0.3353667	
		YN	3	0.4777	
		SD	3	0.6935667	
	Significance			0.069	
		PA	N	b	a
Maslinic acid	Duncan [a]	AH	3	0.1293667	
		JX	3	0.2593	0.2593
		HN	3	0.261	0.261
		ZJ	3	0.2724667	0.2724667
		YN	3	0.3012333	0.3012333
		SD	3		0.5311667
	Significance			0.351	0.151
		PA	N	a	
Beta-amyrin	Duncan [a]	HN	3	0.1107667	
		YN	3	0.2237667	
		ZJ	3	0.2700667	
		JX	3	0.33	
		AH	3	0.4285333	
		SD	3	0.4975333	
	Significance			0.077	
		PA	N	b	a
Ursolic acid	Duncan [a]	ZJ	3	0.1121667	
		YN	3	0.1289	0.1289
		AH	3	0.1635667	0.1635667
		JX	3	0.2103667	0.2103667
		HN	3	0.2597667	0.2597667

		SD	3		0.3371333
	Significance			0.157	0.054
Fatty acids c30		PA	N	a	
	Duncan [c]	JX	2	0.0942	
		AH	2	0.10395	
		ZJ	3	0.1240333	
		HN	3	0.2007	
		SD	3	0.2520667	
		YN	2	0.28035	
	Significance			0.128	

Table. S3 The significance analysis of fatty acids c26, c28, primary alcohols c30, oleanolic acid, and fatty acids c29 waxes (Subset of alpha = 0.05. PA is production area. Duncan [a], Duncan [b], and Duncan [d] represent harmonic mean sample size = 3.00, 2.769, and 2.571, respectively).

		PA	N	a	
Fatty acids c26	Duncan [b]	JX	3	0.2049667	
		ZJ	3	0.3129	
		SD	3	0.3521333	
		HN	3	0.3568333	
		AH	3	0.4490667	
		YN	2	0.8475	
	Significance			0.077	
		PA	N	b	a
Fatty acids c28	Duncan [b]	JX	3	0.1113667	
		HN	3	0.1904333	0.1904333
		AH	2	0.4065	0.4065
		SD	3	0.4462	0.4462
		ZJ	3	0.5497333	0.5497333
		YN	3		0.5886667
	Significance			0.053	0.075
		PA	N	b	a
Primary alcohols c30	Duncan [d]	HN	3	0.0000	
		SD	3	0.0000	
		ZJ	3	0.0000	
		JX	3	0.0390	0.0390
		AH	2	0.0647	0.0647
		YN	2		0.4360
	Significance			0.738	0.054
		PA	N	b	a
Oleanolic acid	Duncan [a]	HN	3	0.1104333	
		ZJ	3	0.1379	
		AH	3	0.2376	0.2376
		JX	3	0.3026667	0.3026667
		SD	3	0.3210667	0.3210667
		YN	3		0.4135
	Significance			0.068	0.115
		PA	N	b	a
Fatty acids c29	Duncan [b]	AH	3	0	
		SD	3	0	
		ZJ	2	0.0745	
		JX	3	0.0859667	
		HN	2	0.08915	
		YN	2		0.21555

	Significance	0.101	1

Table. S4 93 kinds compounds with significant pharmacological effects and flavors (19 metabolites from the Jiangxi source were significantly higher than those from other with yellow highlighted).

Formula (NO.)	Class	KEGG	Library	JX1	JX2	JX3	JX4	YN	HN	SD
C20H28O4	Phenols	C21818	BioDeepDB	93743046.61	62706110.03	8844677.088	55097944.57	55606991.88	98420677.8	10720481.06
C6H6O3	Phenols	C02814	BioDeepDB	20137473.22	31770803.34	28868936.26	26925737.61	28965040.46	32903987.89	39256854.84
C7H6O4	Phenols	C00628	BioDeepDB	10474671.36	9907713.875	11202460.31	10528281.85	40032752.63	2585404.313	10382866.56
C8H11NO2	Phenols	C04227	HMDB	3632757.879	2572785.45	9148754.456	5118099.262	7150686.399	2541774.018	6051708.545
C21H24FN3O4	Quinolines and derivatives	C07663	HMDB	5022517.116	9335491.106	13606780.44	9321596.221	7855655.06	2169459.34	12149339.18
C23H22O6 (M_1)	Flavonoids	C07593	MoNA	40855069.35	24938141.06	17444515.84	27745908.75	8529675.97	2714088.94	20853463.24
C8H10O4	Phenols	C05576	BioDeepDB	9746055.623	7204232.401	8390926.924	8447071.649	3497646.546	9534881.979	5065190.933
C8H8O3	Phenols	C00755	NaturalProducts	85006701.46	104297560.4	91679422.84	93661228.23	83198446.59	58430843.59	142556037
C20H20O5	Isoflavonoids	C15509	BioDeepDB	25524150.47	24728773.63	15268069.9	21840331.33	16150217.94	13960766.67	22049612.17
C27H30O16 (M_2)	Flavonoids	C12634	BioDeepDB	26360405.87	2492951.758	2422505.47	10425287.7	564628.4104	4114764.306	5841366.914
C10H10O3	Phenols	C02666	MoNA	20074267.88	16566957.15	6810549.147	14483924.72	8588429.203	14755400.22	17326990.07
C6H5NO3	Phenols	C00870	MoNA	1024308.528	2400694.877	89382691.1	30935898.17	249457508.8	59544595.12	46818608.57
C8H11NO (M_3)	Phenols	C16570	Metlin	148757748.7	86902298.32	193163741	142941262.7	102703561.5	118909528.3	119898675
C9H10O	Phenols	C16930	BioDeepDB	5876018.344	4457471.084	17754182.12	9362557.184	6249877.63	5688542.31	4674321.965
C10H12O2	Phenols	C10469	HMDB	9461312.846	10494648.97	14595672.27	11517211.36	19574238.38	21841452.66	19275330.85
C8H8O4 (M_4)	Phenols	C06672	BioDeepDB	51600024.05	37805552.8	51092785.78	46832787.54	13376768.29	10018284.9	20069693.89
C10H7NO3	Quinolines and derivatives	C01717	MoNA	12871840.25	15751233.8	5371283.239	11331452.43	10843476.45	8436770.739	6703929.08
C9H6O3	Coumarins and derivatives	C09315	MoNA	18111399.84	16354944	21741355.56	18735899.8	20191426.09	15241877.41	19159798.79
C27H30O16 (M_5)	Flavonoids	C05625	Metlin	81160353.32	34650997.92	114369902.6	76727084.6	41419074.96	8530616.576	55510130.1
C15H10O6	Flavonoids	C05903	HMDB	4903304.689	2552797.154	3847913.555	3768005.133	6512429.292	9614146.455	6269867.887
C15H10O5	Flavonoids	C14314	BioDeepDB	1589522.443	2043775.128	3502666.934	2378654.835	4967402.01	2661663.052	3223485.408
C20H20O6	Phenols	C21191	BioDeepDB	249313966.8	150531977.5	170611052.1	190152332.2	186095493.4	102226202.7	380549349.2
C22H24O11	Flavonoids	C16422	BioDeepDB	13476733.36	7817245.215	7941613.624	9745197.401	5167629.783	8343138.435	12958621.18

Formula	Class	KEGG	Library	JX1	JX2	JX3	JX4	YN	HN	SD
C21H20O10	Isoflavonoids	C09126	OTCML	2530696.666	955073.9272	4984555.3	2823441.965	4193988.141	4657904.611	9836760.531
C27H30O14	Flavonoids	C12628	NaturalProducts	1256441.721	24624.64616	371618.7515	550895.0395	263413.6393	184718.6894	327632.4611
C8H11NO2	Phenols	C03758	MoNA	23839111.16	60395410.1	43582593.73	42605705	93056407.38	45160290.55	53841914.22
C15H10O6	Flavonoids	C01514	Metlin	2550709.433	1641501.584	1090534.979	1760915.332	1724425.964	2044191.138	1630317.733
C15H10O8 (M_6)	Flavonoids	C10107	Metlin	521151881.4	524637846.7	300337132.3	448708953.5	522641103.7	262737032.1	513175326.2
C16H12O6	Flavonoids	C04293	HMDB	10388708.89	8036403.634	1375277.84	6600130.122	4892333.578	6083589.555	1604036.437
C10H12O2	Phenols	C10453	MoNA	8373106.039	7518820.264	12559348.95	9483758.418	10573593.59	11607911.7	12062539.47
C16H14O5	Flavonoids	C00786	BioDeepDB	58109764.29	49874634.14	28093172.73	45359190.39	26446485.97	37585103.03	76466174.28
C16H12O5	Flavonoids	C01470	NaturalProducts	16851117.32	17857803.22	18231017.26	17646645.93	18574606.9	19430077.47	16293826.2
C16H14O5 (M_7)	Isoflavonoids	C16190	BioDeepDB	578315411.9	483367250.3	131589938.3	397757533.5	185128137.9	490876923.4	284067129.5
C28H42O2	Phenols	C14155	BioDeepDB	36634.46404	23233374.92	4368023.197	9212677.528	4426228.131	2434831.287	15815095.78
C24H29NO9	Morphinans	C16577	BioDeepDB	48521530.68	44285932.41	6451554.002	33086339.03	342572.7552	94013311.81	6514451.324
C21H22O10	Flavonoids	C16407	BioDeepDB	11307959.93	885012.8792	71368.85525	4088113.887	26274.77523	3787055.381	3188338.519
C15H12O4	Flavonoids	C09827	MoNA	2932112.06	2433734.911	2172565.377	2512804.116	2023577.39	24041265.45	3322578.564
C9H8O2	3,4-dihydrocoumarins	C02274	HMDB	1411616.523	1244568.107	5293726.328	2649970.319	6878878.158	2387268.836	5538090.938
C9H7NO2	Quinolines and derivatives	C05637	BioDeepDB	2278860.936	3955850.67	8225823.135	4820178.247	4735835.504	3607840.489	3897421.061
C16H17NO3	Morphinans	C11785	Metlin	3163109.691	3988709.553	1219151.033	2790323.426	3966714.995	3436434.371	2284138.099
C9H6O4	Coumarins and derivatives	C09263	MoNA	5016639.835	9511600.595	3356977.41	5961739.28	5014880.294	6919264.931	4275080.576
C9H6O2 (M_8)	Coumarins and derivatives	C05851	HMDB	41149384.27	20118775.15	52915444.41	38061201.28	23315420.15	11269069.87	16258421.83
C15H12O6	Flavonoids	C00974	BioDeepDB	4348486.079	1219566.151	1999212.262	2522421.497	2588888.516	1718787.49	2562309.489
C16H12O6	Flavonoids	C10098	NaturalProducts	32332728.12	5027416.096	223726991.4	87029045.2	129047884.1	66846518.08	175753088.6
C12H8O4	Coumarins and derivatives	C01557	MoNA	9631594.221	10073003.59	7769317.229	9157971.678	8109422.728	6614480.18	9725403.19
C16H12O8	Flavonoids	C12633	HMDB	904591.8021	1359494.898	3929970.707	2064685.802	7095333.784	1315699.875	4282500.928
[C21H21O12]+	Flavonoids	C12138	NaturalProducts	12504105.53	3296615.701	476634.7381	5425785.322	239357.7633	3135155.249	833214.0124
C9H13NO3	Phenols	C00788	BioDeepDB	10886899.19	11732987.76	10141644.27	10920510.4	20456602.28	3578012.095	9083932.564

Formula	Class	KEGG	Library	JX1	JX2	JX3	JX4	YN	HN	SD
C15H14O6	Flavonoids	C03648	BioDeepDB	89562.28044	108865.6302	2478447.796	892291.9023	3311077.025	110108.5387	2515125.424
C21H20O11	Flavonoids	C01750	MoNA	2488176.65	507044.9582	469699.6978	1154973.769	361636.8792	217502.3214	1079948.237
C8H8O5	Phenols	C05616	BioDeepDB	1243703.011	4534087.582	11709388.04	5829059.544	11475467.04	2287253.815	9392504.445
C7H6O4	Phenols	C00196	NaturalProducts	15256679.06	9266030.554	7385439.475	10636049.7	8568599.754	3745207.323	10254343.55
C21H20O10 (M_9)	Flavonoids	C01460	NaturalProducts	195490003.1	70807404.69	39684950.95	101994119.6	52163288.29	63466280.57	123922258.9
C14H12O3	Phenols	C03582	MoNA	37643527.72	12620270.13	20481380.73	23581726.2	27595303.62	17716973.24	17461524.98
C16H14O6 (M_10)	Flavonoids	C09756	NaturalProducts	138207017.4	123175651.8	32187316.48	97856661.87	54291859.02	32012624.08	97009963.94
C15H14O8	Flavonoids	C05909	BioDeepDB	887170.2597	1155733.271	2497088.907	1513330.813	13531879.16	1885878.842	4192009.525
C8H8O4 (M_11)	Phenols	C03198	BioDeepDB	67008764.47	63044246.4	21686604.38	50579871.75	35887893.8	13298938.12	28782627.75
C4H4O5	Organic acid	C00036	BioDeepDB	340270.8222	487276.3645	671873.2374	499806.808	1196045.721	481292.766	429944.6081
C6H6O2	Phenols	C01751	BioDeepDB	7221004.528	2736733.561	1673430.808	3877056.299	2453115.739	6433818.204	1241342.5
C15H10O5	Flavonoids	C06563	NaturalProducts	2922035.997	3584238.036	5902765.186	4136346.406	8888837.991	4554879.215	6118854.579
C8H8O5	Phenols	C05580	HMDB	169479.0498	132629.1942	1448623.868	583577.3707	1025188.642	904125.3975	1355455.175
C16H14O5	Flavonoids	C05334	MoNA	102803.9917	140933.7048	399580.1906	214439.2957	513638.5054	120493.6248	1063316.097
C16H12O6 (M_12)	Isoflavonoids	C10520	BioDeepDB	196107109.5	36221094.42	88122052.89	106816752.3	61386891.41	11662675.84	11440233.19
C8H10O2	Phenols	C06044	BioDeepDB	751456.0544	710899.2345	1335636.793	932664.0271	2305534.51	4329776.609	1022862.819
C15H10O7	Flavonoids	C00389	NaturalProducts	667977.6349	495591.6914	1867357.91	1010309.079	993221.3125	3416487.779	2874851.559
C21H24O10	Flavonoids	C01604	NaturalProducts	3537575.674	1939051.807	3374253.156	2950293.546	2428321.742	1515364.972	3840385.631
C8H8O3	Phenols	C00642	BioDeepDB	7199128.931	13477102.88	7881111.073	9519114.296	8031534.199	7785361.937	8029507.435
C20H18O5	Flavonoids	C10422	BioDeepDB	4953783.062	1520797.911	895952.5467	2456844.506	6184969.004	288527.5599	20650225.03
C21H20O11 (M_13)	Flavonoids	C12249	NaturalProducts	192268021.6	87519154.76	44701083.03	108162753.1	26701022.97	86955349.93	70213776.02
C16H22O8	Flavonoids	C00761	BioDeepDB	2820727.02	1675887.855	557885.8228	1684833.566	523465.8812	1456383.335	456166.9656
C15H12O6 (M_14)	Flavonoids	C05631	NaturalProducts	525364499.9	415769583.5	62862049.66	334665377.7	90504033.43	274863320.3	116585549.6
C15H12O6	Flavonoids	C01378	BioDeepDB	6952894.035	2934712.258	54642.15011	3314082.814	297472.8143	3394513.208	456221.8915
C25H26O12	Flavonoids	C16224	BioDeepDB	4442465.45	4274140.359	1912998.153	3543201.32	1702083.429	2082440.167	1409733.879

Formula	Class	KEGG	Library	JX1	JX2	JX3	JX4	YN	HN	SD
C7H12O4	Organic acid	C02656	NaturalProducts	1103364.281	984273.4343	108999.7258	732212.4803	451648.6416	3726145.981	75905.45958
C3H4O3	Organic acid	C00022	BioDeepDB	216825484.1	71692756.01	497328156.4	261948798.8	112114561.4	216576949.2	452690967.2
C16H14O6	Flavonoids	C01709	MoNA	5358719.315	5604202.149	12158310.58	7707077.347	11407806.19	5538635.304	12811278.81
C14H20O7 (M_15)	Phenols	C06046	BioDeepDB	17618052.05	25859811.43	18859790.36	20779217.95	4735144.307	3554165.03	27264941.52
C6H5BrO (M_16)	Phenols	C14453	HMDB	21842042.01	24619555.09	5842065.095	17434554.06	7623680.588	19406215.16	9179138.383
C28H32O14	Flavonoids	C12629	BioDeepDB	8299306.297	7704777.891	2612409.686	6205497.958	2609418.197	3004735.326	3184878.27
C21H20O11 (M_17)	Flavonoids	C03951	BioDeepDB	54619392.07	34326352.06	46895362.84	45280368.99	28711368.67	22223346.42	18258280.22
C15H14O7	Flavonoids	C12127	BioDeepDB	14938255.06	8015908.001	552011.6894	7835391.582	2456231.006	1241686.993	2740376.766
C15H14O7	Flavonoids	C05906	BioDeepDB	512105.6321	574449.6963	6556226.95	2547594.093	5147317.047	991550.5002	4127284.68
C15H10O6 (M_18)	Isoflavonoids	C12134	BioDeepDB	12568271.9	39705888.8	8508788.977	20260983.23	5207866.128	7764680.375	8088065.431
C15H10O3 (M_19)	Flavonoids	C01495	OTCML	67283064.13	26017641.99	6859519.499	33386741.87	3362888.052	26375273.76	23552350.41
C20H19NO5	Protopine alkaloids	C05189	BioDeepDB	479824.6305	672264.0407	469925.1589	540671.2767	395528.7368	57234.52041	379278.548
C17H12O6	Coumarins and derivatives	C06800	Metlin	16108044.83	10351224.51	31095363.2	19184877.51	44468089.35	33659591.88	27751465.98
C16H12O5	Flavonoids	C01562	BioDeepDB	563814.6639	468082.5167	9962.107719	347286.4294	4126.806224	10358181.44	32547.43858
C6HCl5O	Phenols	C02575	NaturalProducts	1482661.35	736023.062	36311.8634	751665.425	386644.4218	260540.8472	178606.494
C16H12O6	Flavonoids	C16227	BioDeepDB	2761394.651	2288286.254	1167721.922	2072467.609	355849.8547	3082078.114	1970479.153
[C20H18NO4]+	Protoberberine alkaloids and derivatives	C00757	BioDeepDB	9779494.591	1694863.625	3118260.994	4864206.403	2854472.017	1343181.422	1225312.892
C21H18O12	Flavonoids	C03515	BioDeepDB	4680868.355	4127720.455	1164037.207	3324208.673	742700.6304	1821428.884	2676597.477
